# Chondroitin sulfate proteoglycan 4 regulates zebrafish body axis organization via Wnt/planar cell polarity pathway

**DOI:** 10.1371/journal.pone.0230943

**Published:** 2020-04-02

**Authors:** Yen-Hua Lee, Koichi Kawakami, Wei-Chun HuangFu, I-Hsuan Liu

**Affiliations:** 1 Department of Animal Science and Technology, National Taiwan University, Taipei, Taiwan; 2 Laboratory of Molecular and Developmental Biology, National Institute of Genetics, Mishima, Shizuoka, Japan; 3 Department of Genetics, SOKENDAI (The Graduate University for Advanced Studies), Mishima, Shizuoka, Japan; 4 The Ph.D. Program for Cancer Biology and Drug Discovery, College of Medical Science and Technology, Taipei Medical University, Taipei, Taiwan; 5 School of Veterinary Medicine, National Taiwan University, Taipei, Taiwan; 6 Research Center for Developmental Biology and Regeneration Medicine, National Taiwan University, Taipei, Taiwan; University of Colorado Boulder, UNITED STATES

## Abstract

Pericellular and extracellular proteoglycans play an important role in modulating morphogen gradients and signal transductions. Chondroitin sulfate proteoglycan 4 (Cspg4) is a membrane spanning proteoglycan expressed in immature progenitor cells and cancer cells. Cspg4 participates in cellular events such as proliferation, migration and signal transduction, and these events are generally important for embryo development. In this study, we characterized Cspg4 for its roles in zebrafish embryonic development. Our results demonstrated that *cspg4* was maternally expressed from 0 to 3 hours post fertilization (hpf) and expressed in the anterior and posterior embryo end after 9 hpf. Knocking-down *cspg4* resulted in a shorter anterior-posterior axis than control embryo, which could be rescued by co-injecting *wnt11* mRNA suggesting that Cspg4 regulates body axis organization through modulating the Wnt/planar cell polarity signaling pathway. In addition, overexpressing *cspg4* caused cyclopia. The Cspg4 transmembrane domain mutant embryo phenocopied the global over-expression of *cspg4* mRNA and led to cyclopia with a very low penetrance. Our results demonstrated that the quantitatively and spatially accurate distribution of Cspg4 is critical for body axis and midline development during gastrulation.

## Introduction

Chondroitin sulfate proteoglycan 4 (Cspg4), also known as neuron glial antigen 2 (NG2), high molecular weight melanoma-associated antigen (HMW-MAA), or melanoma chondroitin sulfate proteoglycan (MCSP), is a membrane-spanning glycoprotein with a large extracellular domain that can link glycosaminoglycan (GAG) chains [1]. The extracellular domain includes three sub-domains: the N-terminal laminin G-type domain, the central domain containing multiple chondroitin sulfate acceptor sites and collagen V and VI binding sites and the juxtamembrane domain with at least two proteolytic sites [2].

Cspg4 is expressed in many immature progenitor cells such as oligodendrocyte progenitor cells [3], mesenchymal stem cells [4], chondroblasts [5], pericytes [6] and melamoma cells [7]. Previous studies demonstrated that Cspg4 promotes cell proliferation, modulates melanoma cell migration, interacts with the extracellular matrix and participates in signal transductions [8–11]. It is known that chondroitin sulfate GAGs (CS-GAGs) regulate cell division and are required for the maintenance of pluripotency and differentiation commitment in embryonic stem cells [12, 13]. However, not only the CS-GAGs but also the core protein of Cspg4 affects cell behavior. The core protein of Cspg4 has high binding affinity to basic fibroblast growth factor (bFGF) and platelet-derived growth factor-AA (PDGF-AA) [9], and in turn promotes cell proliferation and migration. For example, the aortic smooth muscle cells isolated from CSPG4 null mice do not proliferate and migrate in response to PDGF-AA [10]. Blocking of CSPG4 with the CSPG4-specific antibody on triple-negative breast cancer (TNBC) cells inhibits cell growth, adhesion, and migration [11]. In addition to acting as a co-receptor, Cspg4 also interacts with the extracellular matrix proteins such as tenascin, laminin, type II collagen, type V collagen and type VI collagen [8]. In glioma cells, CSPG4 anchors on collagen VI and thus enhances migration [14]. Furthermore, Cspg4 acts as a co-receptor of integrin α4β1 and activates the Rho GTPase protein family and FAK pathway [15, 16].

During gastrulation, cell fate specification, adhesion and migration lead to convergent extension that the embryo narrows along the medial-lateral axis and extends along the anterior-posterior axis. Anterior-posterior axis formation begins from the mid-blastula stage in the zebrafish embryo. At this stage, β-catenin signaling induces the expressions of Nodal related genes *ndr1* (*sqt*) and *ndr2* (*cyc*) in marginal blastomeres [17]. These Nodal ligands diffuse toward the animal pole and produce a signaling gradient that is essential for mesendoderm formation and patterning [18]. Marginal cells receiving low level Nodal signaling become notochord (posterior axial mesoderm), whereas high level Nodal signaling is required for prechordal plate (anterior axial mesoderm) formation [19]. Once the prechordal plate cells are specified, these cells begin to internalize and migrate toward the animal pole [20]. This migration is regulated by signaling pathways such as PDGF-PI3K, Wnt/planar cell polarity (PCP) pathway and STAT3 signaling [21]. The polarity and shape of cells are the key factors that regulate the movements of convergent extension during gastrulation. At the onset of gastrulation, the activation of PDGF-PI3K signaling induces polarization of the prechordal plate cells. In the absence of PI3K signaling, cell migration is slowed, but is still highly directed [22]. Furthermore, *wnt11* mutants have abnormal extension of axial tissue and midline defects, and the movement of prechordal plate cells is less oriented and slower than in the wild-type embryo [23, 24].

Accumulating evidence shows the roles of proteoglycans and GAGs on the morphogen gradient and signaling. A heparan sulfate proteoglycan Knypek is known to interact with Wnt11 and control the convergent extension cell movement during zebrafish gastrulation [25]. Also the hyaluronan synthase (*has2*) is reported to be involved in the convergent extension during zebrafish gastrulation [26]. In this study we characterized zebrafish Cspg4 during early embryogenesis and provided evidence to show that Cspg4 contributes to the body axis organization during gastrulation via the Wnt/PCP pathway.

## Materials and methods

### Zebrafish maintenance

The AB strain wild-type zebrafish and *cspg4*^*em1twu0405*^ fish were kept in the automatic recirculation aquatic system with 14 hours light and 10 hours dark light cycle per day. Systemic water temperature was maintained at 28°C. Embryos were collected immediately after spawning and kept 28.5°C [27]. All experimental procedures on animals were approved by the Institutional Animal Care and Use Committee (IACUC) of National Taiwan University (NTU105-EL-00016).

### Temporal and spatial expression pattern of *cspg4*

To validate the temporal expression pattern of *cspg4* in early embryonic development, total RNA of zebrafish embryos at 0, 3, 6, 9, 12, 16, 20, 24, 48, 72, 96 and 120 hours post fertilization (hpf) was extracted using the GENEzol reagent (Geneaid Biotech, Taipei, TW) according to the manufacturer’s instruction. The cDNA of zebrafish mRNA was synthesized using the SuperScript III First-Strand Synthesis System (Invitrogen, Carlsbad, CA, USA) according to the manufacturer’s instruction. After cDNA was synthesized, polymerase chains reactions were performed with the *cspg4* primer pair to verify its temporal expression pattern (F: GGT AGA CAG ACA GCC AAC CT; R: TTT GTT GTC CGA CA G TGC TG).

*In situ* hybridization was used to observe the spatial expression profile of *cspg4*, *ntl* and *myoD* as previous described [28]. Briefly, zebrafish embryos were treated with 0.003% N-Phenylthiourea (PTU; Sigma-Aldrich, St. Louis, MO, USA) after 20 hpf. Before staining, chorion was removed and embryos were fixed in 4% paraformaldehyde (PFA; Sigma-Aldrich) at 4°C overnight. The specimen was then transferred to methanol (Sigma-Aldrich) and stored at -20°C. Before hybridization, samples were rehydrated, treated with proteinase K (GMbiolab Co. Taichung, TW), re-fixed by PFA, and pre-hybridized with hybridization buffer at 65°C. Then, the sample was incubated with 50 ng of probe in 200 μL hybridization buffer at 68°C overnight. After washing several times, the specimen was blocked at room temperature for 3 hours and incubated with 1/5000 Anti-Digoxigenin-AP Fab fragments (Roche, Basel, CH) at 4°C overnight. The NBT/BCIP Stock Solution (Roche) was used to detect hybridization.

### Molecular cloning

The primers used in molecular cloning were designed using the NEBuilder Assembly Tool (New England Biolabs, Ipswich, MA, USA). To construct pT7-cspg4^stop-FLAG-IRES-dsRed plasmid, cDNA sequence of *cspg4* was cloned using one primer pair to clone the 5’ end (*cspg4*_1–3200_F: GAG AAG ATC TCG AGC TCA AGC TTC GAT CAT TTT CAT CCA CGT C; *cspg4*_1–3200_R: TCA ATA AGC GCT GAT CAT TTC TGA CAA CGT TAA ATG). The other primer pair was used to clone the 3’ end of *cspg4* (*cspg4*_3201–7072_F: TGT CAG AAA TGA TCA GCG CTT ATT GAC CAC TG; *cspg4*_3201–7072_R: GGG CCC GCG GTA CCG TCG ACC TAC TTG TCG TCA TCG TCT TTG TAG TCa acc cag tac tgg ttc tt). These primers contained 25 base-pair overlapping region (underline) with pT7-IRES2-DsReds vector [29]. Two *cspg4* fragments and the linearized vector were ligated using the Gibson Assembly Master Mix (New England Biolabs) according to the manufacturer’s instruction.

To generate pT7-wnt11^stop-HA-IRES -dsRed and pT7-pdgfaa-IRES-dsRed plasmid, *wnt11* and *pdgfaa* were cloned gene specific primer-pairs containing overlapping region (underline) with pT7-IRES2-DsRed and HA-tag coding sequences (lower case) (wnt11_F: CGA GCT CAA GCT TCG AAT TCT CAG ACA GTC CGT GGT GTA TC; wnt11_R_HA: TAC CGT CGA CTG CAG AAT TCt caa gcg taa tct gga aca tcg tat ggg taC TTG CAG ACG TAT CTC TCG) (pdgf-aa_F: CGA GCT CAA GCT TCG CAC GCT CCG CTC CGC GCT; pdgf-aa_R: TAC CGT CGA CTG CAG TTT GAG AAA CAA ACA CAA TAA AAT GTA TTC GCT TTA TTA GTA TAA GAG TTA CTG TAG ATT GAG TTT TGC TGC). After *pdgfaa* and *wnt11* were cloned from zebrafish cDNA, these fragments were assembled with linearized pT7-IRES2-DsRed vector by Gibson Assembly Master Mix (New England Biolabs) according to the manufacturer's instruction. Plasmid sequences were confirmed by sequencing service (Center for Biotechnology, National Taiwan University, Taipei, TW).

### Morpholinos and mRNA microinjection

To knockdown Cspg4 protein function, standard control morpolino (CCT CTT ACC TCA GTT ACA ATT TAT A) and morpholinos against the second exon-intron splicing site (GAT GCT GCA AAC ACA GAG CAG AGA A) or translational starting site (GGC TCT CAT CTT GCC ACT TTA GTC C) were purchased (Gene Tools LLC, Philomath, Oregon, USA). All morpholinos were dissolved in RNase free water to make 2 mM stock. To make the template for mRNA synthesis, the control plasmid (pT7-IRES2-DsRed) and plasmids containing T7 promoter and *cspg4*, *pdgf-aa* or *wnt11* were linearized and purified. Messenger RNA was synthesized by mMESSAGE mMACHINE T7 Transcription Kit (Invitrogen) and purified by LiCl precipitation. Morpholino and mRNA were mixed with 0.25% phenol red before microinjection. Embryos were collected immediately after spawning. Morpholino and mRNA were injected into one-cell stage embryos. After injection, embryos were kept in 28.5°C. The morphology of embryo was observed and recorded by microscope (Leica Z16-APO) at 10 and 24 hpf. The anterior-posterior angle and body length were measure by ImageJ [30].

### Alcian blue staining

Zebrafsih embryos were anesthetized at 3 days post fertilization (dpf) and fixed in 4% PFA at least overnight at 4°C. A modification of previous reported Alcian blue staining protocol was used to stain the cartilage of larval zebrafish [31]. Fixed samples were stained using the 0.02% Alcian blue blue 8GX (Sigma-Aldrich) in 70% ethanol (Sigma-Aldrich) and 0.37% HCl at 4°C overnight. After staining, samples were washed 3 times in 70% ethanol and 0.37% HCl for 30 minutes. Then, the samples were rehydrated stepwise in 70%, 50% and 25% ethanol/ PBS. To enhance optical clarity, samples were washed 3 times in 2% KOH, and bleached with 3% H_2_O_2_ and 1% KOH for one hour. After that, samples were washed in 2% KOH for three times and left until clear. When the samples were clear as desired, they were transferred to glycerol for imaging.

### Generation of Cspg4 mutant line by CRISPR/Cas9 system

CRISPR/Cas9 system was used to generate the Cspg4 transmembrane domain mutant fish. Cas9 encoding plasmid pCS2-hSP-Cas9 (Addgene #51815) was linearized by *Not*I digestion and purified by phenol chloroform method. One μg of linearized DNA template was used to synthesize Cas9 mRNA with the mMESSAGE mMACHINE SP6 transcription Kit (Invitrogen). The sgRNA were constructed as previously described [32]. The guide-RNA target sequence before Cspg4 transmembrane domain was designed using CHOPCHOP website [33] (https://chopchop.rc.fas.harvard.edu/index.php). The DNA oligo with T7 promoter sequence (TAA TAC GAC TCA CTA TA), target sequences excluding the PAM (GGA GAT CTT AAA CAC AAC CG) and the overlapping region with the constant sequence (GTT TTA GAG CTA GAA ATA GCA AG) was purchased (Invitrogen). Gene-specific oligo (100 μM) was annealed to 100 μM constant oligo nucleotide (AAA AGC ACC GAC TCG GTG CCA CTT TTT CAA GTT GAT AAC GGA CTA GCC TTA TTT TAA CTT GCT ATT TCT AGC TCT AAA AC) by slow cooling from 95°C to room temperature in 1 hour. Then, T4 DNA polymerase (Takara Bio, Kusatsu, Shiga, JP) was used to fill-in the nucleotide. After that, double strand DNA oligo was purified by PCR purification kit. After purification, DNA template was used to synthesize sgRNA with MEGAshortscript T7 Transcription Kit (Invitrogen) according to the manufacturer’s manual. For injection, a mixture containing 25 ng sgRNA and 250 ng Cas9 mRNA in 0.2 M KCl (Sigma-Aldrich) and 0.25% phenol red was injected in to one-cell stage wild type zebrafish embryo.

### Genotyping of transmembrane domain mutant line *cspg4*^*em1twu0405*^

To extract genomic DNA, adult zebrafish were anesthetized in 0.025% 3-Aminobenzonic acid ethyl ester (Tricain) in system water. After that, 50% of caudal fins were collected by razor. The removed caudal fin was incubated with 30 μL alkaline lyse reagent (25 mM NaOH, 0.2 mM EDTA, pH 12) at 95°C for 45 minutes. The samples were then cooled at 4°C and 30 μL 40 mM Tris-HCl (pH 5) was added to neutralize DNA sample. To validate the genotype, PCR was performed with the DNA samples and *cspg4* primer pair (F: GGT AGA CAG ACA GCC AAC CT; R: TTT GTT GTC CGA CA G TGC TG). PCR products were analyzed by 1.5% agarose electrophoresis. The wild-type PCR product size was 927 bp and 1202-bp PCR product was mutant fish.

### Western blot

To characterize mutated Cspg4, HEK293T cells were seeded at 4 × 10^5^ cells/ 6 cm^2^ dish and cultured using 10% fetal bovine serum (FBS, Hyclone, Logan, UT, USA) Dulbecco’s modified Eagle’s medium (DMEM, Gibco, Grand Island, NY, USA). After cultured for 24 hours, culture medium was change to FBS free DMEM and transfected with pT7-cspg4^stop-FLAG-IRES-dsRed or mutated pT7-mcspg4^stop-FLAG-IRES-dsRed by using *Trans*IT-X2 Transfection Reagent (Mirus Bio, Madison, WI, USA). Twenty four hours after transfection, culture medium was collected and concentrated by Amicon Ultra-15 30K (Millipore Co., Billerica, MA, USA) according to manufacturer’s instruction. The total protein of cells was extracted with lysis buffer (50 mM Tris HCl, pH 7.4, with 150 mM NaCl, 1 mM EDTA, and 1% TRITON X-100). The BCA protein assay kit was used to quantify the protein concentration. Sixty or 20 μg total protein from culture medium or cell lysate was separate by 8% SDS-polyacrylamide gel electrophoresis (PAGE) and subsequently transfer onto a PVDF membrane. The membrane was blocked and probed with a 1:2000 dilution of DDDDK tag antibody (ab21536, Abcam, Cambridge, MA, USA), and a 1:5000 dilution of goat anti-rabbit antibody (PerkinElmer Inc., Waltham, MA, USA) was used as secondary antibody. The signal was detected by Chemiluminescence Reagent (Immobilon Western, Millipore Co., Billerica, MA, USA).

### Immunoprecipitation

To investigate the interaction between Cspg4 and Wnt11, HEK-293T cells were transfected with pT7-cspg4^stop-FLAG-IRES-dsRed or pT7- wnt11^stop-HA-IRES-dsRed, or co-transfected with both plasmids as above described. After total protein was extracted by lysis buffer, Anti-FLAG M2 Magnetic Beads (Sigma-Aldrich) were used to precipitate flag-tagged Cspg4 protein according to the manufacturer’s instruction. After immunoprecipitation, samples were separated by 12% SDS-PAGE or 3~8% tris-acetate gel for detecting HA-tagged Wnt11 or flag-tagged Cspg4, respectively. Protein was transfer onto PVDF membranes and blocking with 5% bovine serum albumin (Sigma-Aldrich). After blocking, membrane was hybridized with HA-Tag (C29F4) Rabbit mAb (#3724, Cell Signaling Technology, Danvers, MA, USA) or DDDDK tag antibody (ab21536, Abcam), and a 1:5000 dilution of goat anti-rabbit antibody (PerkinElmer Inc.) was used as secondary antibody. The signal was detected by Chemiluminescence Reagent.

### Statistical analysis

All values in this study were present as mean ± standard deviation. Student’s *t*-test, one-way ANOVA with Tukey multiple comparisons or Kruskal–Wallis test followed by Dunn’s multiple comparisons were performed by using Prism 6 software (Graphpad software, San Diego, CA, USA). *P* value less than 0.05 was considered as statistically significant.

## Results

### Cspg4 is expressed during zebrafish embryogenesis

**Fig 1:**
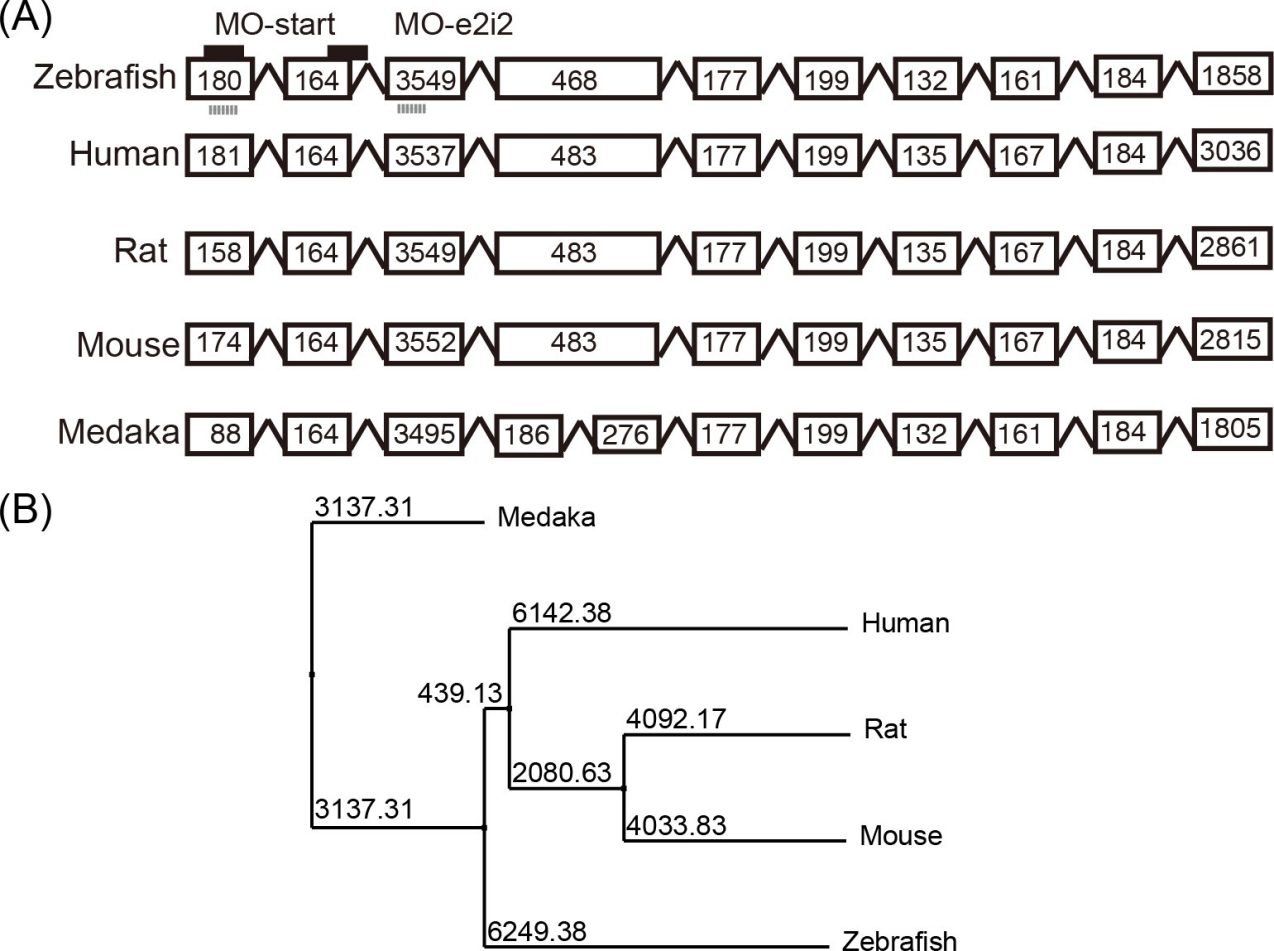
The genome structure and protein sequence of zebrafish *cspg4* are similar to other vertebrates. (A) The genome structure of *cspg4* in zebrafish (ENSDART00000112782) compared to human (ENST00000308508), rat (ENSRNOT00000023326.3), mouse (ENSMUST00000035661) and medaka (ENSORLT00000002266.1). Scheme demonstrates the target sites of splicing (MO-e2i2) and translational blocking (MO-start) morpholino (black line) in zebrafish. Gray lines indicate primer pairs used to evaluate pre-mRNA processing. (B) The protein sequence of Cspg4 among zebrafish (UniProt # E7FCN9), rat (UniProt # F1LS79), human (UniProt # Q6UVK1), mouse (UniProt # Q8VHY0) and medaka (UniProt # H2L8T3) were analyzed by Jalview2 software with PAM250 matrix and the Neighbour Joining method [37].

The protein structures of Cspg4 and their respective functions had been characterized in other species such as rat, mouse and human [1, 10, 34]. To elucidate the genome structure and protein function, we first compared the exonal structure and protein sequence of zebrafish Cpsg4 with other species. Zebrafish *cspg4* had 10 exons and the exonal structure was similar to mammalian CSPG4 (Fig 1A). Phylogenetic analysis also suggested that zebrafish Cspg4 was highly conserved throughout evolution (Fig 1B). We predicted the functional domains of wild-type zebrafish Cspg4 using the InterPro website [35]. Like other vertebrates [36], zebrafish Cspg4 was predicted to be a transmembrane proteoglycan, containing a large extracellular domain (2211 amino acids) and a short intracellular domain (68 amino acids). The extracellular domain contained two laminin G domains and 15 chondroitin sulfate proteoglycan repeats.

**Fig 2:**
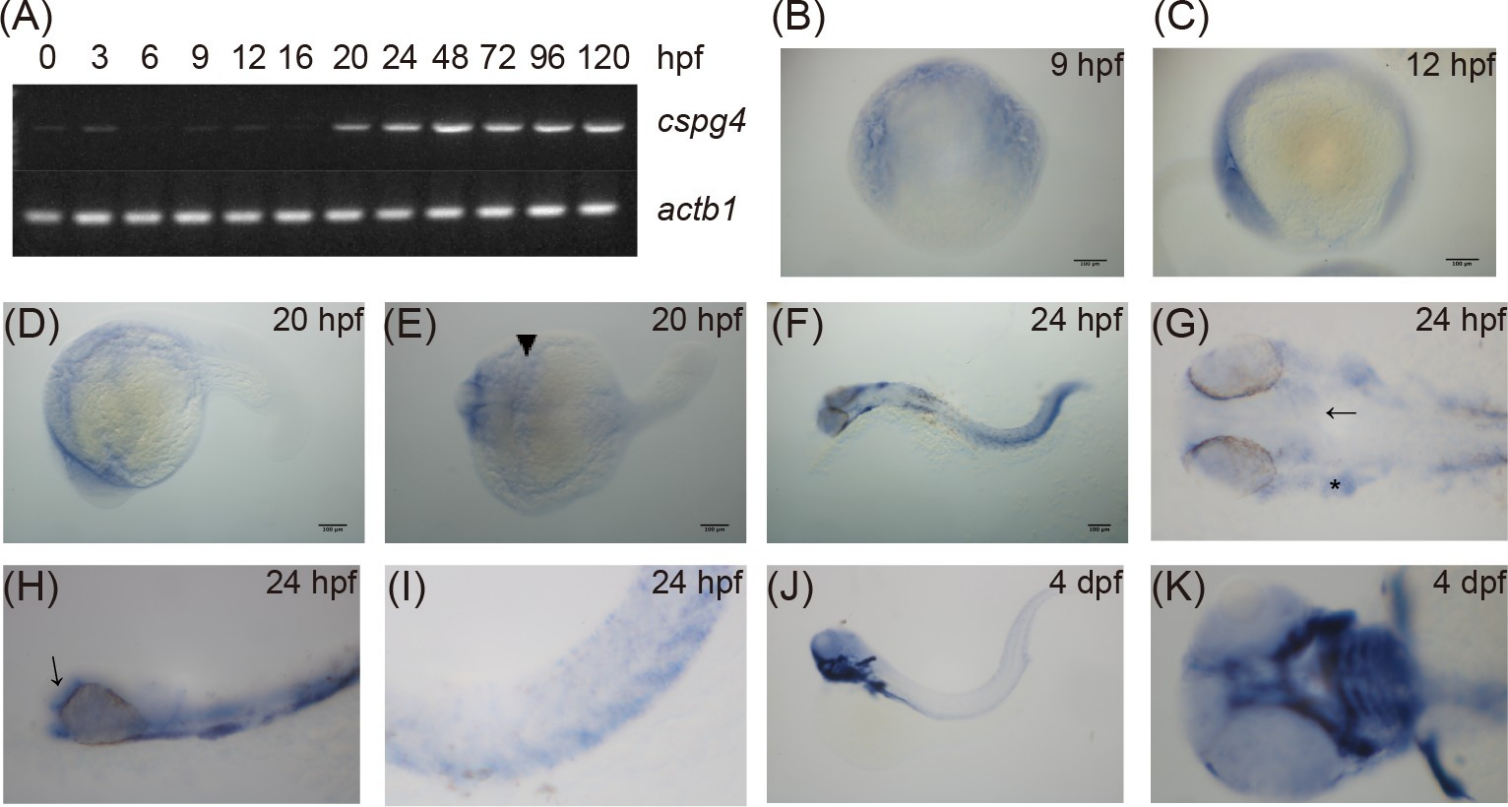
Zebrafish embryo expressed *cspg4* during development. (A) The expression of *cspg4* was detected by RT-PCR from 0 to 120 hpf with *actb1* as loading control. (B-H) Expression pattern of *cspg4* was revealed by whole-mount *in situ* hybridization. At 9 (B) and 12 hpf (C), *cspg4* was expressed in both the anterior and posterior end of the embryos. Lateral view (D) and dorsal view (E) of 20 hpf embryos showed that *cspg4* was mainly expressed in the anterior paraxial and lateral mesoderm (arrow head). (F) *Cspg4* was expressed in head, somite, ventral mesenchyme and tail at 24 hpf. Scale bar represents 100 μm. (G) Cspg4 was expressed in head mesenchyme (arrow) and otic placode (asteridsk). (H) Lateral view of 24 hpf embryo showed that *cspg4* was expressed in the head mesenchyme (arrow) and pharyngeal region. (I) *Cspg4* was expressed in the ventral sclerotome. (J) *Cspg4* expressed in jaw, pectoral fins, and trunk at 4 dpf. (K) Ventral view of a 4 dpf larvae showed that *cspg4* was expressed in pharyngeal cartilage and the pectoral fin.

To evaluate the temporal and spatial expression patterns of *cspg4* during zebrafish embryonic development, RT-PCR and whole-mount *in situ* hybridization were performed. The result of RT-PCR showed that *cspg4* was maternally expressed from 0 to 3 hpf. Maternally deposited *cspg4* mRNA appeared to be cleared before shield stage (6 hpf) and the zygotic *cspg4* was expressed after 9 hpf (Fig 2A). At 9 and 12 hpf, *cspg4* was detected in the anterior and posterior end of the embryo (Fig 2B and 2C). Messenger RNA of *cspg4* was expressed in the anterior paraxial mesoderm and anterior lateral mesoderm at 20 hpf (Fig 2D and 2E). At 24 hpf, *cspg4* mRNA was observed in the otic placode, head mesenchyme (Fig 2F, 2G and 2H), pharyngeal (Fig 2H) ventral sclerotome and ventral mesenchyme (Fig 2F and 2I). *Cspg4* was expressed in pharyngeal arches, pectoral fins and sparsely in somites at 4 dpf (Fig 2J and 2K). These results revealed that *cspg4* was expressed in specific tissues during embryogenesis.

### Zebrafish body axis elongation and pharyngeal cartilage patterning required Cspg4

**Fig 3:**
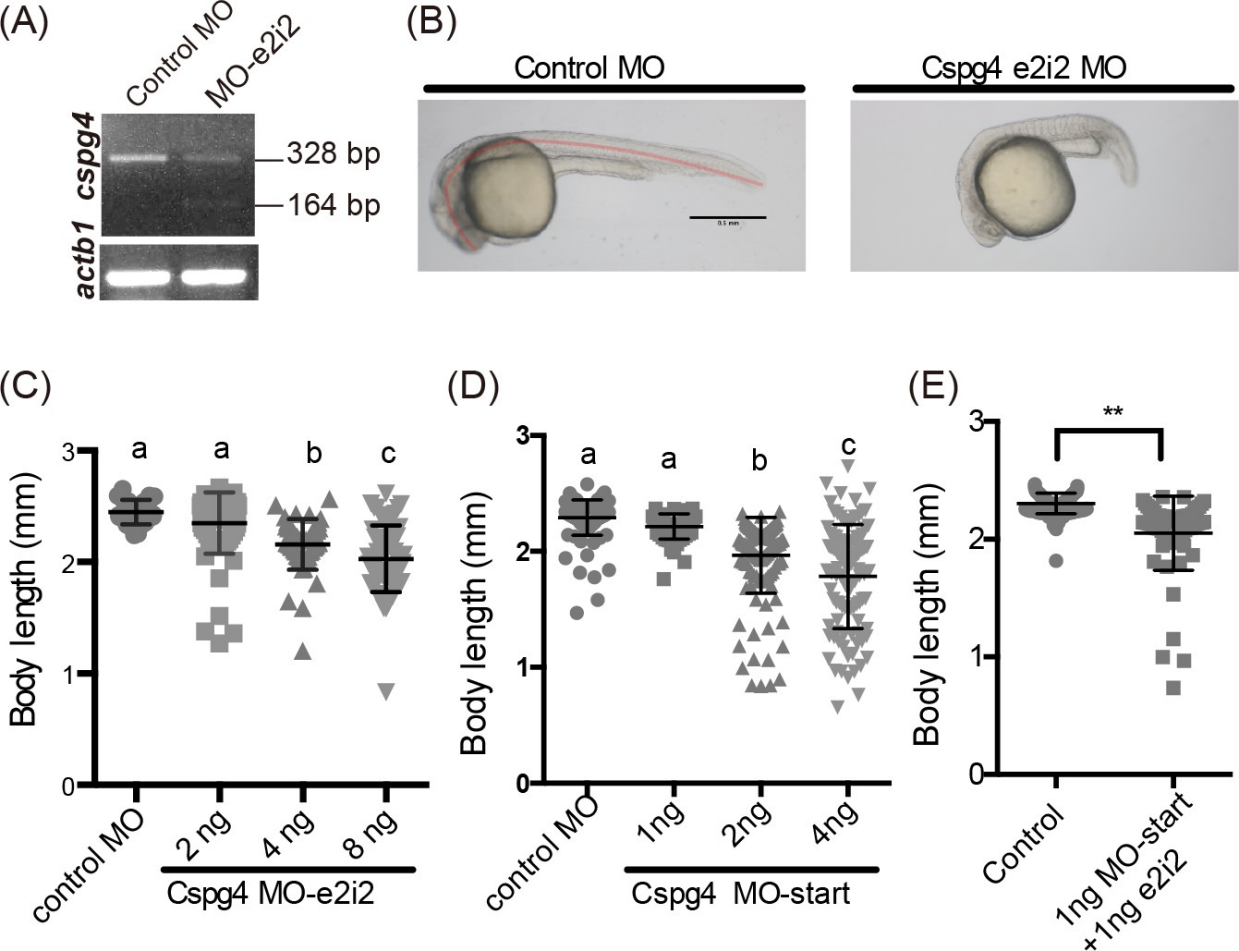
*Cspg4* knockdown led to shorter body length. (A) The 164 bp band indicated the skipping of exon two. (B) *Cspg4* knockdown embryos showed shorter body lengths than control morpholino injected embryos at 1 dpf. The body lengths were measured by using the freehand tool in ImageJ (as labeled by the red line). (C) *Cspg4* splicing blocking morpholino shortened body length in a dose-dependent manner (each dot represents one embryo; Control-MO, n = 26; 2 ng MO-e2i2, n = 78; 4 ng MO-e2i2, n = 53; 8 ng MO-e2i2, n = 67). (D) Knockdown *cspg4* using translational blocking morpholino also shortened body length at 1 dpf (control-MO, n = 140; 1 ng MO-start, n = 60; 2 ng MO-start, n = 139; 4 ng MO-start, n = 105). (E) Combining ineffective doses of *cspg4* MO-e2i2 and MO-start shortened the body length at 1 dpf (control-MO, n = 103; 1 ng MO-start +1 ng MO-e2i2, n = 67). In panel (C and D) the significant difference between groups were tested by ANOVA followed by Tukey’s multiple comparison. Different letters (a, b or c) indicate a significant difference between groups (*p* ≤ 0.05). Student’s *t*-test was used to analyze data in panel (E). ** indicates *p* ≤ 0.01. Data are presented as mean ± SD. Scale bar represents 0.5 mm.

To elucidate the potential roles of Cspg4 during embryogenesis, two anti-sense morpholino oligos targeting zebrafish *cspg4* were used to knockdown gene expression. A translational blocking morpholino targeting the translational starting site (MO-start), and a splicing blocking morpholino (MO-e2i2) targeting the exon-intron boundary were designed and tested (Fig 1A). To test whether MO-e2i2 interfered with pre-mRNA processing, we injected 4 ng MO-e2i2 and performed RT-PCR using primers flanking the first to third exons. The result showed that the mRNA was disrupted by skipping exon 2 resulting in a frameshift and introducing an early stop codon after the 25th amino acid (Fig 3A). After the perturbation of mRNA was confirmed, 2, 4 and 8 ng MO-e2i2 were injected into one-cell stage wild-type zebrafish embryos, while 4 ng of control morpholino was used as a control. We found that *cspg4* knockdown with 4 ng or more of MO-e2i2 resulted in significantly shorter body lengths in a dose-dependent manner (2 ng: 2.35 ± 0.276; 4 ng: 2.16 ± 0.226; and 8 ng: 2.028 ± 0.299 mm) compared to the control group (2.45 ± 0.109 mm) at 1 dpf (Fig 3B and 3C). This shorter body length phenotype was also found in MO-start morphants (1 ng: 2.21 ± 0.108; 2 ng: 1.97 ± 0.327; 4 ng: 1.783 ± 0.449 mm compared to control morphants: 2.29 ± 0.152 mm) (Fig 3D). To further validate the gene function of *cspg4*, we selected several single-guide RNA (sgRNA) targeting the first exon and used CRISPR/Cas9 or CRISPR interference to knockout or knockdown *cspg4*. However, none of these sgRNA knockout or knockdown *cspg4* successfully (S1 Text). Therefore, to eliminate the possibility of unspecific effects by the morpholino injection, we co-injected two different morpholino (MO-e2i2 and MO-start) with ineffective dose. The results showed that combining morpholinos with different target sites also led to the shorter body length phenotype (control-MO: 2.303 ± 0.088; 1 ng MO-start +1 ng MO-e2i2: 2.051 ± 0.315) (Fig 3E) suggesting *cspg4* play a part in body axis organization.

**Fig 4:**
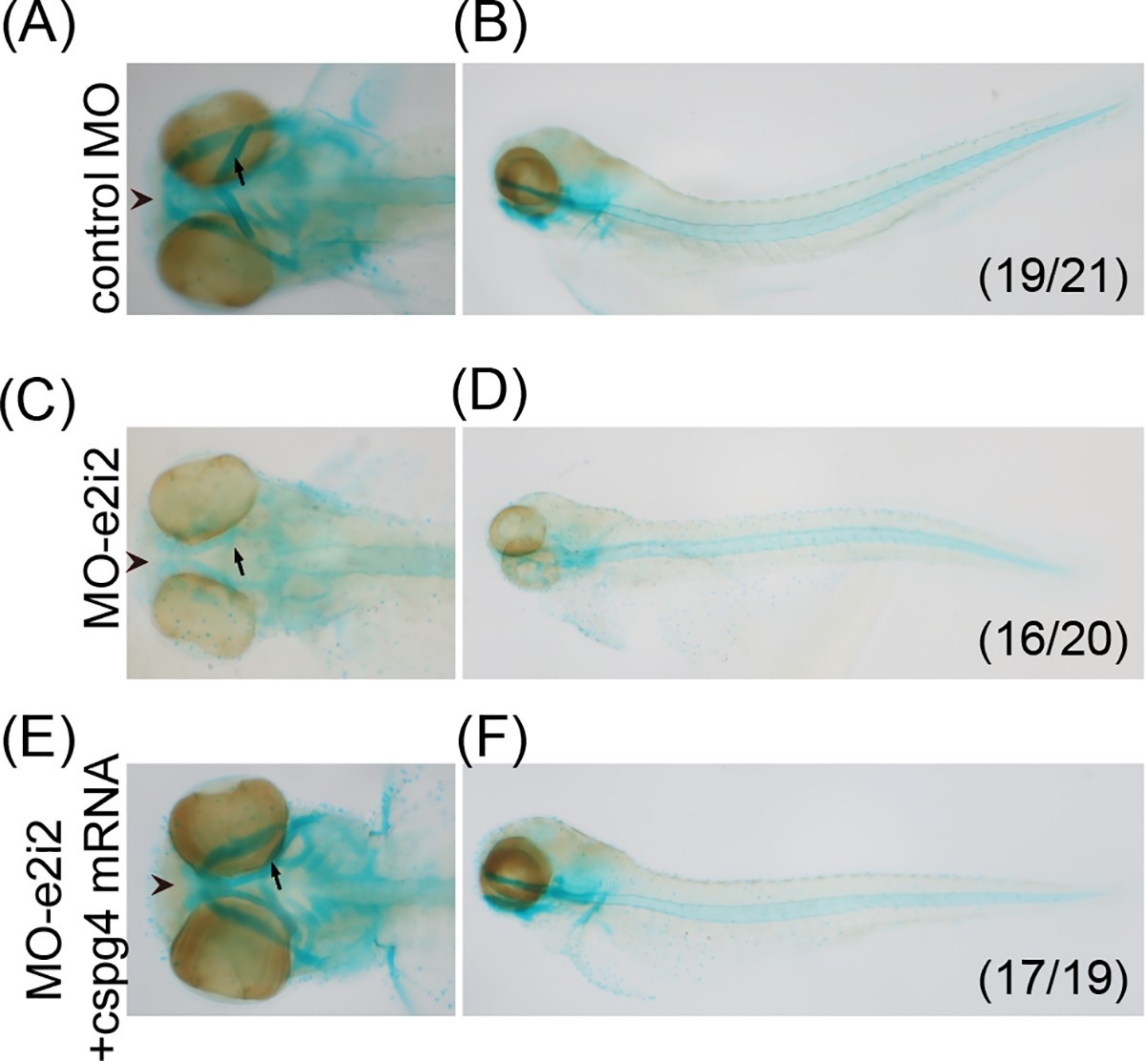
*Cspg4* morphants had abnormal pharygeal cartilage at 3 dpf. The cartilage was stained by using Alcian blue staining. (A, B) The ventral view and lateral view of 4 ng control MO injected embryo at 3 dpf. (C, D) Four ng *cspg4* MO-e2i2 injected morphants had disrupted Meckel’s cartilage (arrow head) and underdeveloped ceratohyal cartilage (arrow). (E, F) The phenotypes of abnormal pharyngeal cartilage development were rescued by co-injecting 250 pg *cspg4* mRNA with 4 ng MO-e2i2. The number in bottom right indicates the penetrance of phenotype.

Furthermore, knocking-down *cspg4* also led to abnormal pharyngeal arch patterns. At 3 dpf, the Meckel’s cartilage and ceratohyal cartilage of 4 ng control-MO injected embryos had formed (Fig 4A and 4B). In contrast, 80% 4 ng *cspg4* MO-e2i2 morphants had underdeveloped Meckel’s cartilage and shorter ceratohyal cartilage when compared to control embryos (Fig 4C and 4D). The deformed pharyngeal cartilage phenotype was rescued by co-injecting 250 pg *cspg4* mRNA with 4 ng MO-e2i2. Most embryos (89.5%) had normal pharyngeal cartilage pattern after co-injecting *cspg4* mRNA with MO-e2i2 (Fig 4E and 4F) suggesting Cspg4 is required for pharyngeal cartilage formation.

**Fig 5:**
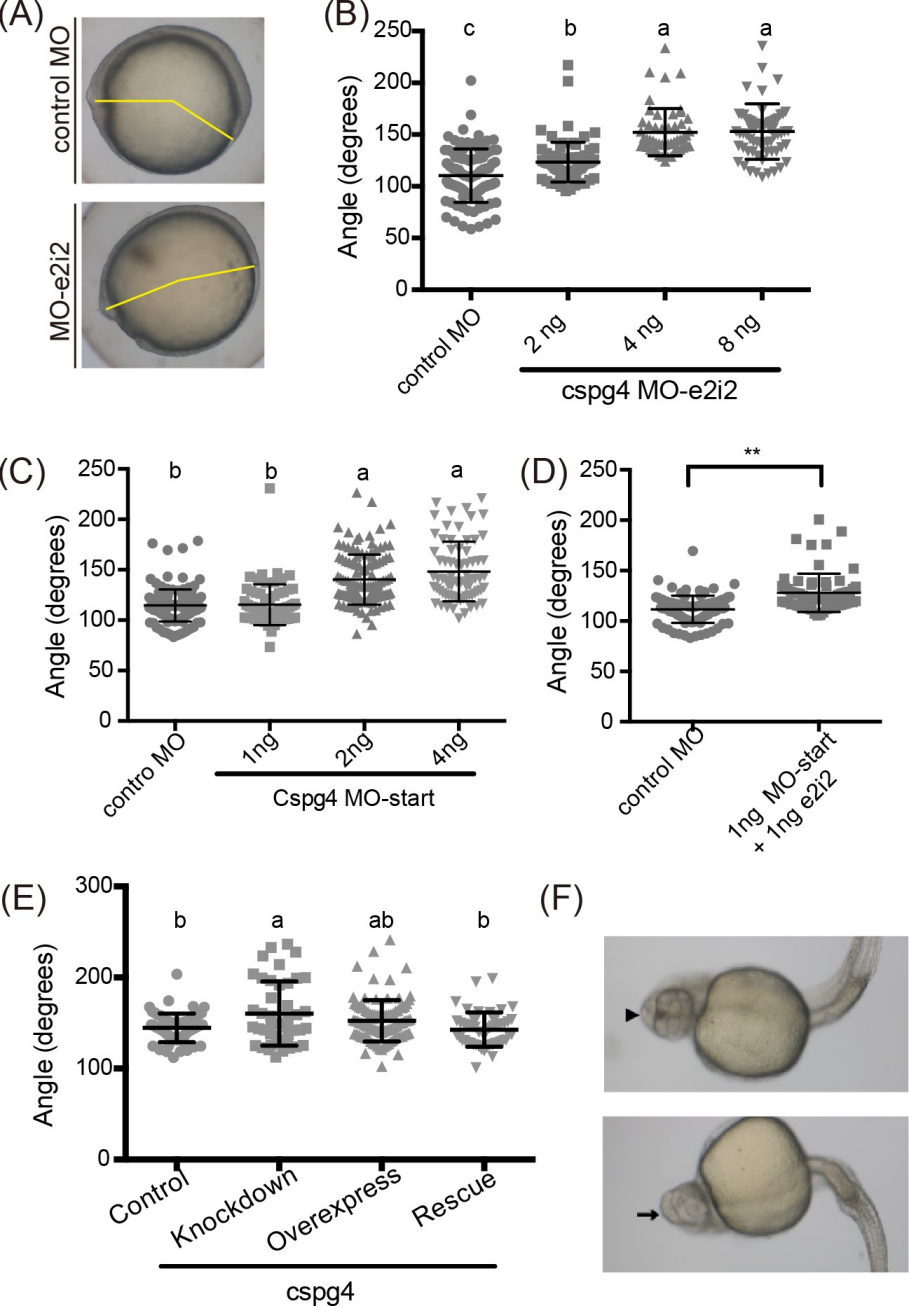
*Cspg4* knockdown led to abnormal body axis formation at tailbud stage. (A) Incomplete body axis organization was caused by *cspg4* knockdown. The degrees of angle between anterior end of embryo and tailbud (labeled by yellow line) were measured by ImageJ. (B) Quantitative results of the angle between anterior end and tailbud were analyzed (each dot represents one embryo. Control-MO, n = 97; 2 ng MO-e2i2, n = 78; 4 ng MO-e2i2, n = 52; 8 ng MO-e2i2, n = 66). (C) Knockdown *cspg4* using translational blocking morpholino also had larger angle between anterior end of embryo and tailbud (control-MO, n = 144; 1 ng MO-start, n = 70; 2 ng MO-start, n = 122; 4 ng MO-start, n = 73). (D) Co-injecting the ineffective dose of MO-e2i2 (1 ng) and MO-start (1 ng) also led to the same phenotype. (E) The angle between anterior end and tailbud was significantly reduced when co-injecting 250 pg *cspg4* mRNA with 4 ng *cspg4* morpholino (control, n = 63; knockdown, n = 43; overexpress, n = 99; rescue, n = 51). (F) About 12% (18/148) of embryos had fused eyes (indicated by arrowhead) or only one eye (indicated by arrow). In figure (B), (C) and (E), the significant difference between groups were tested by ANOVA followed by Tukey’s multiple comparison. Different letters (a, b or c) indicate significant difference between groups (*p* ≤ 0.05). Student’s *t*-test was used in analyzed data in panel (D), ** indicates *p* ≤ 0.01. Data are presented as mean ±SD.

Previous studies demonstrated that gastrulation is a key stage for body axis formation [38–40]. To evaluate body axis formation at the tailbud stage (10 hpf), we measured the angle between the anterior end and tailbud as previously described [41] (Fig 5A). The *cspg4* morphants had a greater anterior-posterior angle in a dose-dependent fashion (2 ng: 123.3 ± 19.25, 4 ng: 152.3 ± 22.83 and 8 ng: 153.0 ± 26.72 degrees) compared to control embryos (110.3 ± 25.80 degrees) (Fig 5B). The *cspg4* MO-start morphants also had the same phenotype in a dose-dependent manner (1 ng MO-start: 115.3 ±20.18; 2 ng MO-start: 140.2 ± 24.97; 4 ng MO-start: 148.2 ± 29.55 degrees compared to 4 ng control morpholino: 114.6 ± 15.91 degrees) (Fig 5C). In order to reduce the non-specific effect of morpholino, we co-injected the *cspg4* MO-e2i2 and MO-start with ineffective doses and found that the angle between anterior end and tailbud were significantly larger compared to 4 ng control morpholino injected group (1 ng MO-start +1 ng MO-e2i2: 128.0 ±19.07; control MO: 111.7 ± 13.42 degrees) (Fig 5D). To further validate that this phenotype is specific to *cspg4* knockdown, 250 pg *cspg4* mRNA was co-injected with MO-e2i2, and the defect in *cspg4* morphants was rescued by co-injecting *cspg4* mRNA (control: 144.6 ± 15.71, *cspg4* MO-e2i2: 160.4 ± 35.18, *cspg4* mRNA rescue: 142.7 ± 18.88 degrees) (Fig 5E). Thus, Cspg4 plays a part in body axis formation. Surprisingly, a small percentage of embryos injected only the *cspg4* mRNA resulted in abnormal eye development (Fig 5F). When embryos had ectopically excessive amounts of *cspg4* mRNA, about 12% (18/148) of embryos had fused eyes or only one eye (Fig 5F). This result suggested the quantitative and/or spatial expression patterns of *cspg4* were also important for zebrafish body axis formation and midline development.

### The location of Cspg4 was important for midline development

**Fig 6:**
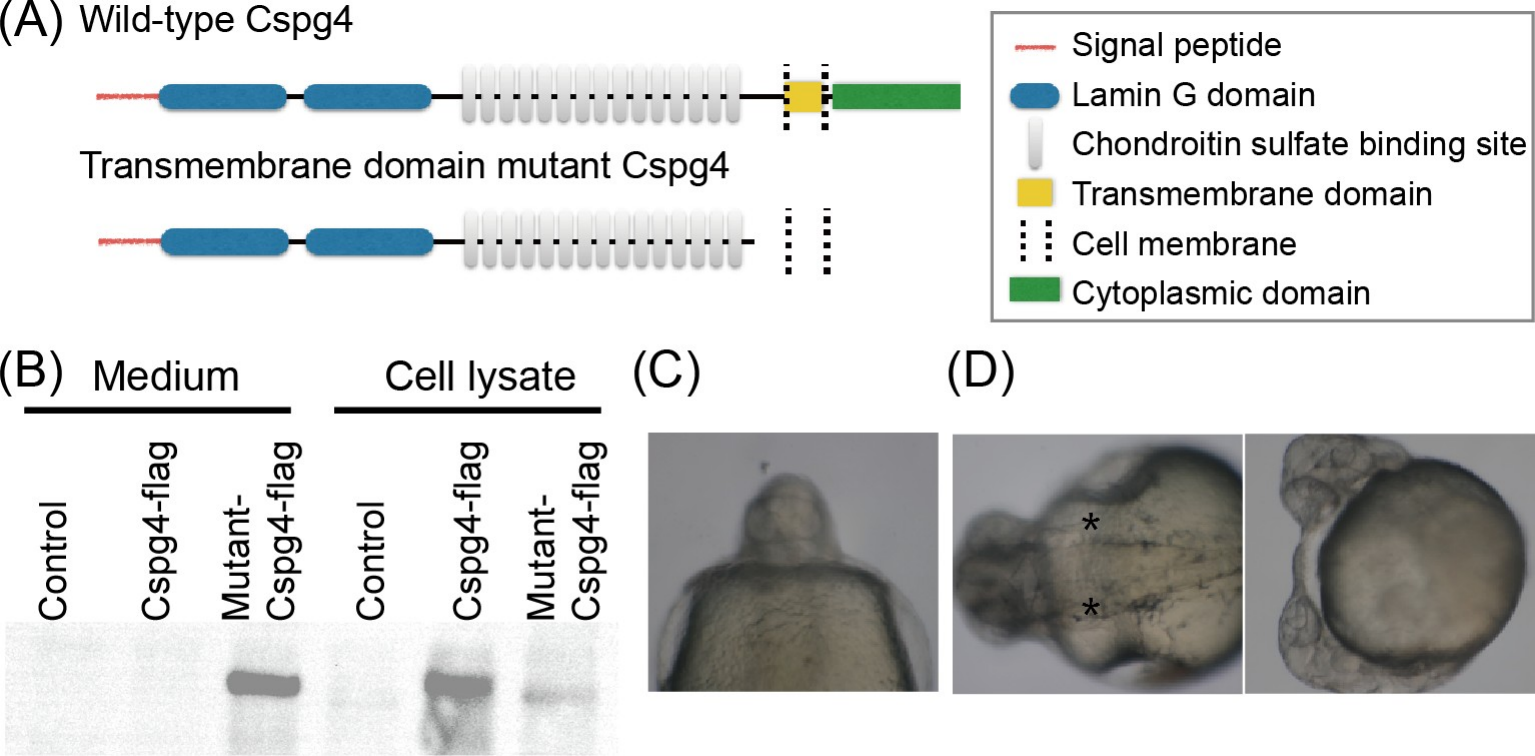
Cspg4 transmembrane domain mutant embryo phenocopied *cspg4* overexpression phenotype. (A) Scheme illustrates wild-type and mutant Cspg4 protein structure. (B) The flag-tag signal of both wild-type and mutant Cspg4 were detected in the cell lysate, but in culture medium the signal of flag-tag was only detected in mutant Cspg4-flag transfected group. (C) Eight percent of *cspg4* ^*em1twu0405*^ embryos were cyclopia. (D) Four percent of *cspg4* ^*em1twu0405*^ embryos showed midline bifurcation (notochord was indicated by asterisk).

Since *cspg4* was expressed in a specific region during early embryogenesis and ectopic overexpression of *cspg4* led to abnormal body axis and midline development, it is likely that the spatial distribution of Cspg4 is critical for zebrafish embryogenesis. To verify whether the abnormal eye development was caused by the ectopic distribution of Cspg4, we generated a Cspg4 transmembrane domain mutant. In the zebrafish, the extracellular region of Cspg4 contains 2 laminin G domains and 15 potential chondroitin sulfate GAG attachment sites as predicted by the InterPro website [35]. The transmembrane region of Cspg4 is between the 2212 to 2237th amino acids that is encoded by the 10th exon (Fig 6A). Thus, a sgRNA was designed targeting the 10th exon and a transmembrane domain mutant fish was created using the CRISPR/Cas9 system. The mutant line *cspg4*^*em1twu0405*^ has a 275 base pair insertion in the 10th exon. The inserted sequence contains 159 bp repeat sequence from the upstream of the sgRNA target site and 110 bp repeat sequence from the downstream of sgRNA target site (S1 Fig). This mutation abolishes the transmembrane domain and introduces a premature stop codon after the 2163th amino acid. To test whether the truncated Cspg4 protein was secreted by cell, the coding sequence of wild-type or mutant *cspg4* were fused with a flag-tag sequence immediately before the stop codon and transfected to HEK-293T cells. The flag-tag signal was detected by western blot. The result showed that, both wild-type and mutant Cspg4 were detected in the cell lysate, but in culture medium the signal of flag-tag was only detected in mutated Cspg4 (Fig 6B). This result indicated Cspg4 in the mutant line is a secretory protein. To observe the phenotype of *cspg4*^*em1twu0405*^, the founder fish (F0) were crossed with AB wild-type zebrafish to generate the heterozygous F1 generation. Heterozygous F1 were inter-crossed and the offspring were genotyped. Like embryos overexpressing *cspg4*, 12% (9/75) mutant embryos had abnormal midline development including 8% fused eyes (Fig 6C) and 4% midline bifurcation (Fig 6D). These results support the notion that location of Cspg4 is important for midline development.

### Cspg4 regulates body axis organization by Wnt11 pathway

**Fig 7:**
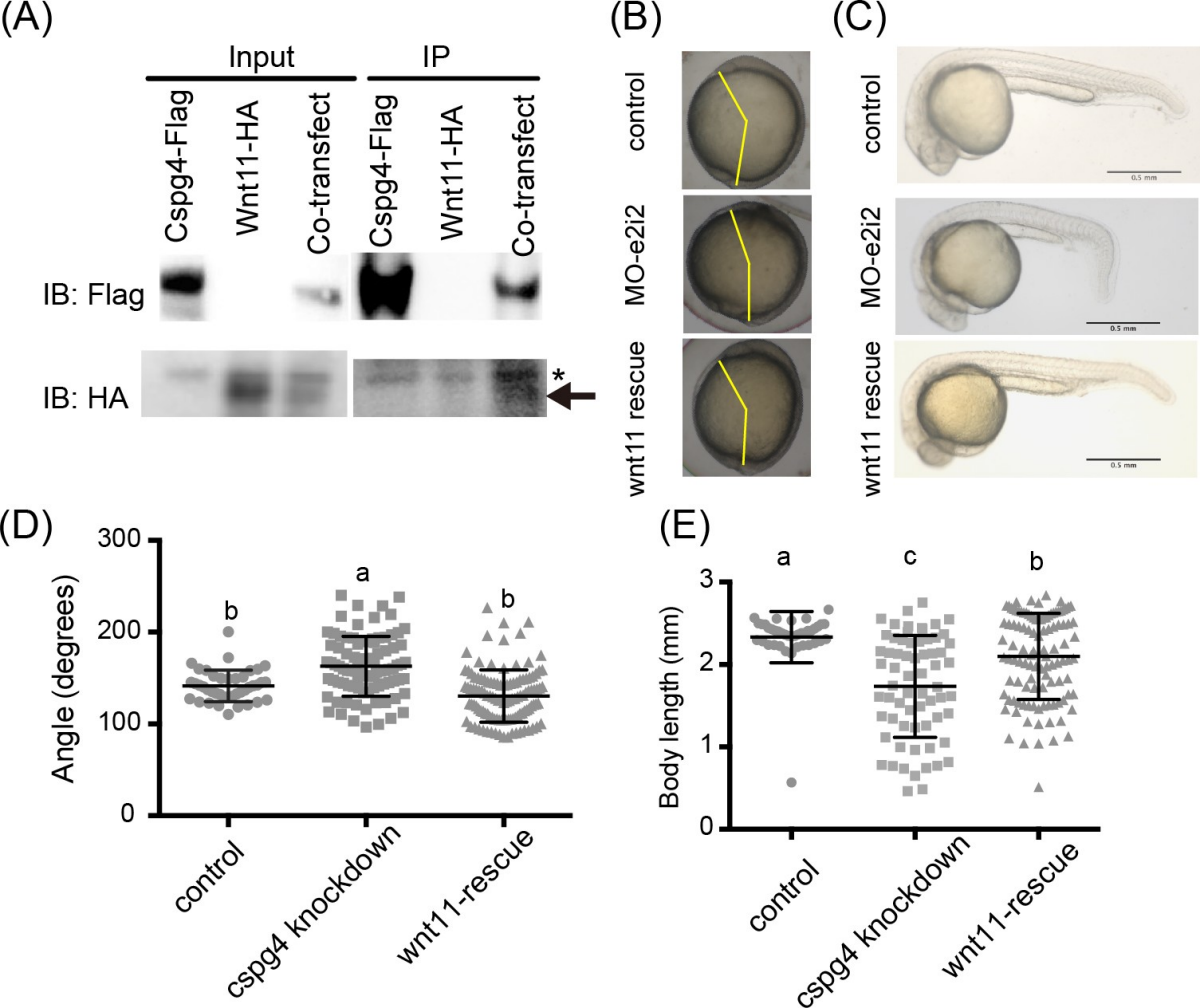
The phenotypes of *cspg4* morphants were rescued by co-injecting *wnt11* mRNA. (A) Wnt11 had binding affinity with Cspg4. Wnt11-HA (indicated by arrow) was co-precipitated with Cspg4-flag. Asterisk mark (*) indicates non-specific band. (B) The abnormal body axis formation phenotype in *cspg4* morphants (4ng MO-e2i2) was rescued by co-injecting 25 pg *wnt11* mRNA. Yellow lines indicate the angle between anterior end and tailbud. (C) Co-injecting 25 pg *wnt11* mRNA with 4 ng *cspg4* morpholino rescued the short body length phenotype. (D) The angle between anterior end and tailbud was reduced in *wnt11* rescue group compared to *cspg4* knockdown group (control, n = 41; *cspg4* knockdown, n = 89; *wnt11* rescue, n = 117). (E) The body length of *wnt11* rescue group at 1 dpf was significantly longer than *cspg4* knockdown group (control, n = 40; *cspg4* knockdown, n = 66; *wnt11* rescue, n = 109). The significant difference was tested using ANOVA followed by Tukey’s multiple comparison. Different letters (a, b or c) indicate significant difference between groups (*p* ≤ 0.05). Data are presented as mean ± SD.

Previous studies demonstrate that both PDGF signaling and Wnt/PCP signaling pathways regulate convergent extension cell movement [22, 23]. Since proteoglycans are known to interact with various morphogens and growth factors such as Wnt and PDGF, it is possible that Cspg4 regulates body axis organization via regulating signal intensity and distribution. The core protein of Cspg4 is known to have binding affinity to PDGF-AA [9]. To test whether Cspg4 participates in the convergent extension via PDGF signaling, mRNA of PDGF-AA was co-injected with *cspg4* MOs and there were no signs of rescue effects (S2 Fig). On the other hand, the *cspg4* knocked-down and overexpressed embryos phenocopied the *wnt11* knocked-down zebrafish embryos [23] and had defects in body axis organization, abnormal pharyngeal cartilage pattern and cyclopia (Figs 4, 5 and 6). To test whether Cspg4 regulates zebrafish early development through Wnt11 pathway, we evaluated the binding affinity between Cspg4 and Wnt11. The flag-tagged Cspg4 and HA-tagged Wnt11 were co-transfected into HEK-293T cells. Wnt11-HA protein was co-immunoprecipitated with Cspg4-Flag (Fig 7A), suggesting that Cspg4 and Wnt11 had direct interaction. Next, we co-injected 25 pg of *wnt11* mRNA with 4 ng of MO-e2i2 to enhance the Wnt11 signal in order to test the rescue effect. When co-injecting *wnt11* mRNA with MO-e2i2, the angle between anterior end and tailbud was significantly smaller than in *cspg4* morphants (control: 141.5 ± 17.14, *cspg4* MO-e2i2: 162.8 ± 32.58, *wnt11* rescued: 130.5 ± 28.47 degrees) at the tailbud stage (Fig 7B and 7D). Furthermore, *wnt11* rescued the short body length phenotype, the body lengths of *wnt11*-rescued group were significantly longer than *cspg4* morphants (2.20 ± 0.48 and 1.59 ± 0.66 mm, respectively) (Fig 7C and 7E).

**Fig 8:**
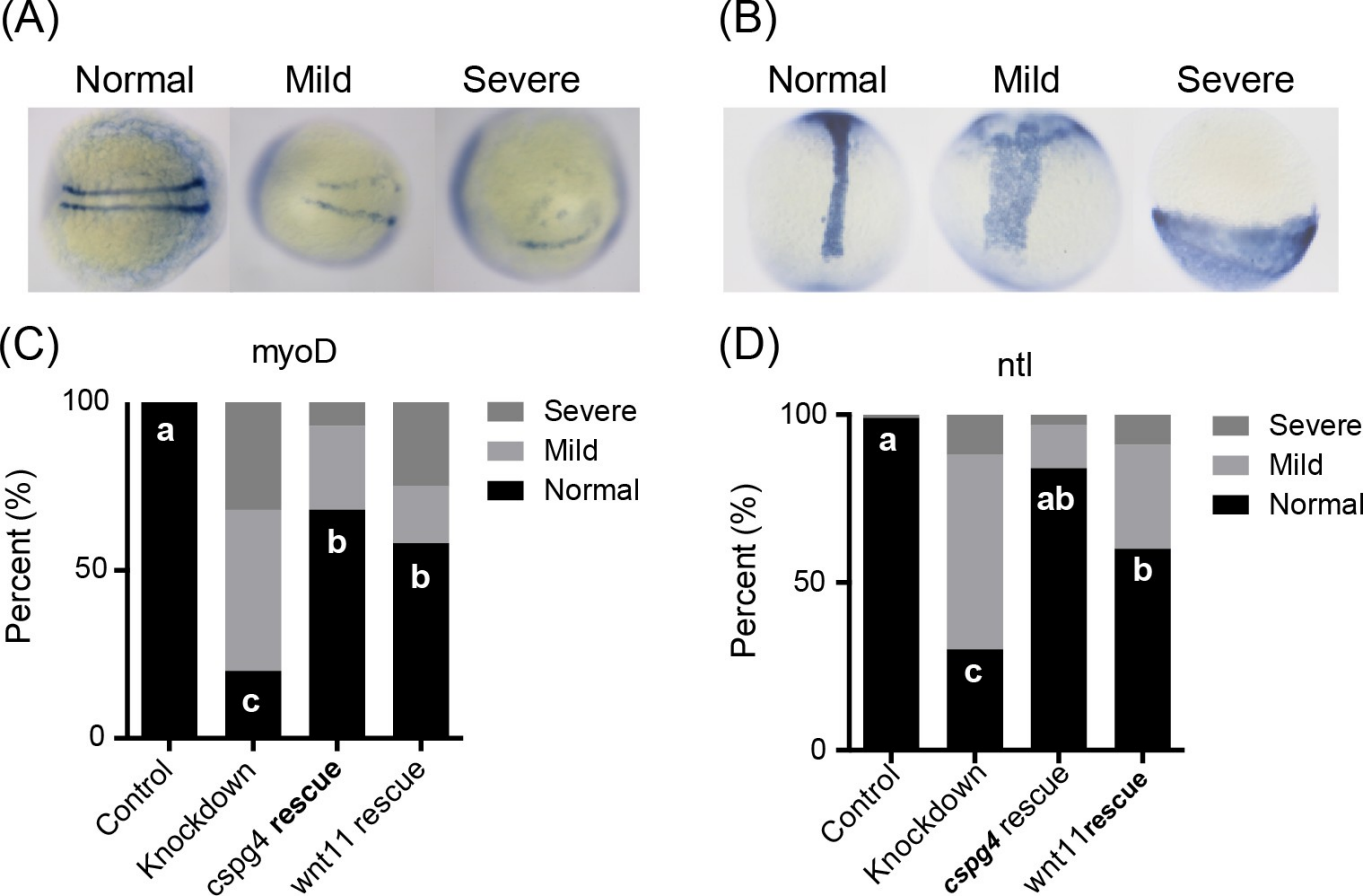
Knockdown of *cspg4* affects paraxial mesoderm marker *myoD* and notochord marker *ntl* expression pattern. (A) *Cspg4* morphants had a wider and shorter (mild) or unilateral (severe) *myoD* expression pattern compared to control morphants (normal) at 12 hpf. (B) The notochord was wider and shorter (mild) or absence (severe) in *cspg4* morphants compared to control morphants (normal) at 12 hpf. (C) The percentage of embryos with abnormal *myoD* expression pattern in each group (control, n = 41; *cspg4* knockdown, n = 40; cspg4 rescue, n = 28; *wnt11* rescue, n = 24). (D) The percentage of embryos with abnormal *ntl* expression pattern in each group (control, n = 73; *cspg4* knockdown, n = 43; *cspg4* rescue, n = 67; *wnt11* rescue, n = 35). The significant difference of normal rate was tested using Kruskal–Wallis test followed by Dunn’s multiple comparisons test. Different letters (a, b or c) indicate significant difference of normal rate between groups (*p* ≤ 0.05).

To verify the body axis organization during development, we observed the expression pattern of the paraxial mesoderm marker gene *myoD* and the notochord marker gene *ntl* at 12 hpf. Compared to control morphants, 47.5% of *cspg4* morphants had a wider and shorter *myoD* expression pattern, while 32.5% *cspg4* morphants had severe convergent defects that expressed *myoD* unilaterally (Fig 8A and 8C). The abnormal *myoD* expression pattern was rescued by co-injecting *cspg4* or *wnt11* mRNA (*cspg4* rescue: mild = 25%, severe = 7%; *wnt11* rescue: mild = 16%, severe = 25%). The *ntl* expression pattern was shorter and wider in about 58% *cspg4* morphants compared to control morphants (1%) and about 12% embryos had no notochord (severe defect) (Fig 8B and 8D). This abnormal *ntl* expression was rescued by co-injecting *cspg4* or *wnt11* mRNA (*cspg4* rescue: mild = 13%, severe = 3%; *wnt11* rescue: mild = 31%, severe = 8%).

In summary, both phenotypes for the anterior-posterior angle at the tailbud stage and the body length at 24 hpf in *cspg4* morphants are rescued by *wnt11* to levels comparable to the control morphants indicating that *cspg4* regulates body axis formation through the Wnt/PCP pathway.

## Discussion

In this study, we characterized zebrafish *cspg4* in early development stages. The genome structure was highly conserved, and the protein structure was predicted to contain a transmembrane domain, two laminin G domains and 15 chondroitin sulfate attaching sites. This prediction is consistent with the study of other species [36]. In other species, Cspg4 has been reported to play a part in regulating cell motility by its CS-GAG chains, extracellular domain or cytoplasmic domain of core protein; for example, the PDZ domain and PKCα phosphorylation site [42–45]. Therefore, it is possible that zebrafish Cspg4 regulates cell motility during gastrulation by these functional domains.

Similar to our results, a previous study using RNA-seq to evaluate the transcriptional profile of different stages of zebrafish embryo development reveals that *cspg4* is weakly expressed before 1000-cell stage (3 hpf) and the expression level is increased after segmentation, the 20~25 somite stage (19~22 hpf) [46]. However, in our study we found that embryos at 9, 12 and 16 hpf faintly expressed *cspg4* mRNA, whereas the expression of *cspg4* before 10 hpf was critical for embryonic development. This difference might due to the sensitivity between RT-PCR and RNA-seq [47]. Previous RNA-seq data also showed that *wnt11* is continuously expressed after blastula-128 cell stage [46], and the spatial expression pattern of *wnt11* [48] overlaps with *cspg4* in paraxial, lateral mesoderm, and somite. These data are also in line with our conclusion that *cspg4* play a role in PCP via Wnt11 during gastrulation.

In our study, we found that loss of *cspg4* during gastrulation impaired body axis organization and ectopic overexpress *cspg4* resulted in shorter body axis and cyclopia. It has been reported that the extracellular GAG hyaluronan and its synthesizing enzyme Has2 are required for lamellipodia formation and dorsal convergence via the Rac1 pathway. Both ectopically expressing Has2 and constitutively active Rac1 lead to shorter body axis and cyclopia, which are caused by axial tissue extension defect [26]. A study of the other CSPG “Decorin” shows that knockdown of Decorin leads to a shorter head-to-tail distance at 2 dpf [49]. On the other hand, overexpressing Decorin at the one-cell stage caused cyclopia at 3 dpf. Consistently, our knockdown and overexpression results phenocopied the Decorin knockdown and overexpression embryos suggesting that these two CSPGs might play redundant roles in zebrafish gastrulation. Accordingly, Cspg4 and Decorin competitively bind to the same extracellular molecule such as collagen VI [8]. Furthermore, it is now widely accepted that functional redundancy like this may strengthen the robustness of a biological system and in turn increase the tolerance of variability in gene regulatory networks. As a result, we observed consistently mid-to-low penetrance of mid-line and body axis organization phenotypes in the *cspg4* morphants or overexpression embryos.

In this study, we generated a Cspg4 transmembrane domain mutant fish *cspg4*^*em1twu0405*^ and demonstrated that ectopically expressed Cspg4 led to abnormal midline development. In addition, this mutant fish has 275 bp insertion, which is longer than the commonly reported sizes of the CRISPR/Cas9 induced indel. In our mutant fish, the inserted sequences seemed to be duplicated from the upstream and downstream of sgRNA target site. It is believed that the large insertion might be caused by aberrant homology-directed repair; for example, the strand invaded to the wrong place during DNA repairing process [50]. However, the exact mechanisms of the large DNA insertion after DNA double strand break still need further investigation.

The non-canonical Wnt/PCP pathway is known to play an important role in convergent extension and midline development. The Wnt11 loss-of-function mutant fish showed abnormal axial extension and midline defect [23]. In our study, co-injecting *wnt11* mRNA partially rescued *cspg4* knockdown embryos suggesting that Cspg4 might regulate body axis organization through the Wnt/PCP pathway.

Zebrafish mutants in Wnt/PCP pathway components had craniofacial malformation. Two Wnt/PCP ligands Wnt5b and Wnt11 had been reported to played different role during pharyngeal cartilage development. Wnt5b mutant had chondrocyte stacking and intercalation problems; Wnt11 mutant had disrupted craniofacial cartilage structure [51]. In our study, *cspg4* morphants also had disrupted pharyngeal cartilage pattern, suggesting the function of Cspg4 in cartilage morphogenesis might be Wnt11 related.

As it is widely agreed that GAGs can shape the morphogen gradients and modulate morphogen signalings via their binding affinities with a wide range of signaling molecules due to their diverse structures [52]. Although there is no other study demonstrates the affinity between chondroitin sulfate and Wnt11. A heparan sulfate proteoglycan “Knypek” had been reported that it potentiates Wnt11 signaling and mediates zebrafish convergent extension cell movement during gastrulation [25]. It is possible that Cspg4 participates in zebrafish gastrulation by interacting with morphogen ligands such as Wnt11 and modulates their distribution and signaling intensity.

Taken together, we characterized Cspg4 during zebrafish embryogenesis and demonstrated that Cspg4 modulates body axis and midline development through Wnt11 signaling.

## Supporting information

S1 Text The efficiency of sgRNAs is low in the first exon of *cspg4*. (PDF)

S1 Fig The DNA sequence of transmembrane domain mutant line *cspg4*^*em1twu0405*^ was aligned to the wild-type cspg4 cDNA sequence (ENSDART00000112782). *Cspg4*^*em1twu0405*^ had 275 bp insertion and the insertion sequences including 6 base pairs repeats sequence of the 6586th to 6591th bp of wild-type *cspg4* cDNA sequence (labeled by yellow box) and 159 bp repeat sequence from its 6116th bp to the 6274th bp of *cspg4* cDNA sequence (labeled by green box) and 110 bp insertion sequence identical to the 6476th bp to the 6585th bp of *cspg4* cDNA. gRNA target sequence was indicated by lower case.
(PDF)

S2 Fig The phenotypes of *cspg4* morphants could not be rescued by co-injecting *pdgfaa* mRNA. The body length at 1 dpf (A) and the angle between anterior end and tailbud at tailed stage. (B) were not rescued by co-injecting *pdgfaa* mRNA.
(PDF)

S1 Raw Images (PDF)
