# Correction: Chondroitin sulfate proteoglycan 4 regulates zebrafish body axis organization via Wnt/planar cell polarity pathway

**DOI:** 10.1371/journal.pone.0270539

**Published:** 2022-06-22

**Authors:** Yen-Hua Lee, Koichi Kawakami, Wei-Chun HuangFu, I-Hsuan Liu

The gene name *wnt11* appears incorrectly throughout the article. The correct gene name is *wnt11f2*.
