# Supplementary material for: Chondroitin sulfate proteoglycan 4 regulates zebrafish body axis organization via Wnt/planar cell polarity pathway
Source: PLoS One. 2020 Apr 2;15(4):e0230943. doi: 10.1371/journal.pone.0230943 (PMC7117731; doi:10.1371/journal.pone.0230943)
Supplement: S1 Text — (PDF) [file pone.0230943.s001.pdf]

S1_text

The efficiency of sgRNAs is low in the first exon of *cspg4*

To generate the *cspg4* mutant zebrafish by using CRISPR/Cas9 system, sgRNAs were designed targeting the exons of *cspg4* (Table1) (S1_Figre1A) and injected into fertilized eggs with Cas9 mRNA. One day after injection, embryos were collected and heteroduplex mobility assay (HMA) was used to validate the efficiency of sgRNAs. The results showed that the sgRNAs targeting the first exon excerted lower efficiency compared to the sgRNA targeting the 10th exon (S1_Figure1 B, C and D). Due to the low efficiency of sgRNAs targeting the first exon, we did not generate any knockout line from the injected founder thus far.

To knockdown cspg4 by an alternative strategy, CRISPR interference was used and several sgRNAs were injected with de-activated Csa9 (dCas9). However, similar to their knockout efficiencies, these sgRNAs were not able to knockdown the expression level of *cspg4* (S1_Figure 1 E, F, G and H). Therefore, we combined the ineffective dose of *cspg4* morpholinos MO-e2i2 and MO-start to reduce the off-target effect of morpholinos since the morpholino with different sequence might not have the same off-target effect.

Table 1. *Cspg4* sgRNA target sequences

Name	Target sequence	Strand
Cspg4_sg_ex1A	GGTGTACTCATGGAGGGGGC	Reverse
Cspg4_sg_ex1B	GGGTGTACTCATGGAGGGGG	Reverse
Cspg4_sg_ex1C	GCAGGGTGTACTCATGGAGG	Reverse
Cspg4_sg_ex1D	TGGGACCTGGAGAGCGTGCA	Reverse
Cspg4_sg_ex1E	GGAGCAGGTATAGGAGTGCC	Reverse
Cspg4_sg_ex1F	GGTATAGGAGTGCCAGGAAG	Reverse
Cspg4_sg_ex1G	CTCCATTGGTGGTCTGGAGC	Reverse
Cspg4_sg_ex1H	AGCAGTCTTACCTCCATTGG	Reverse
Cspg4_sg_ex10	GGAGATCTTAAACACAACCG	Forward

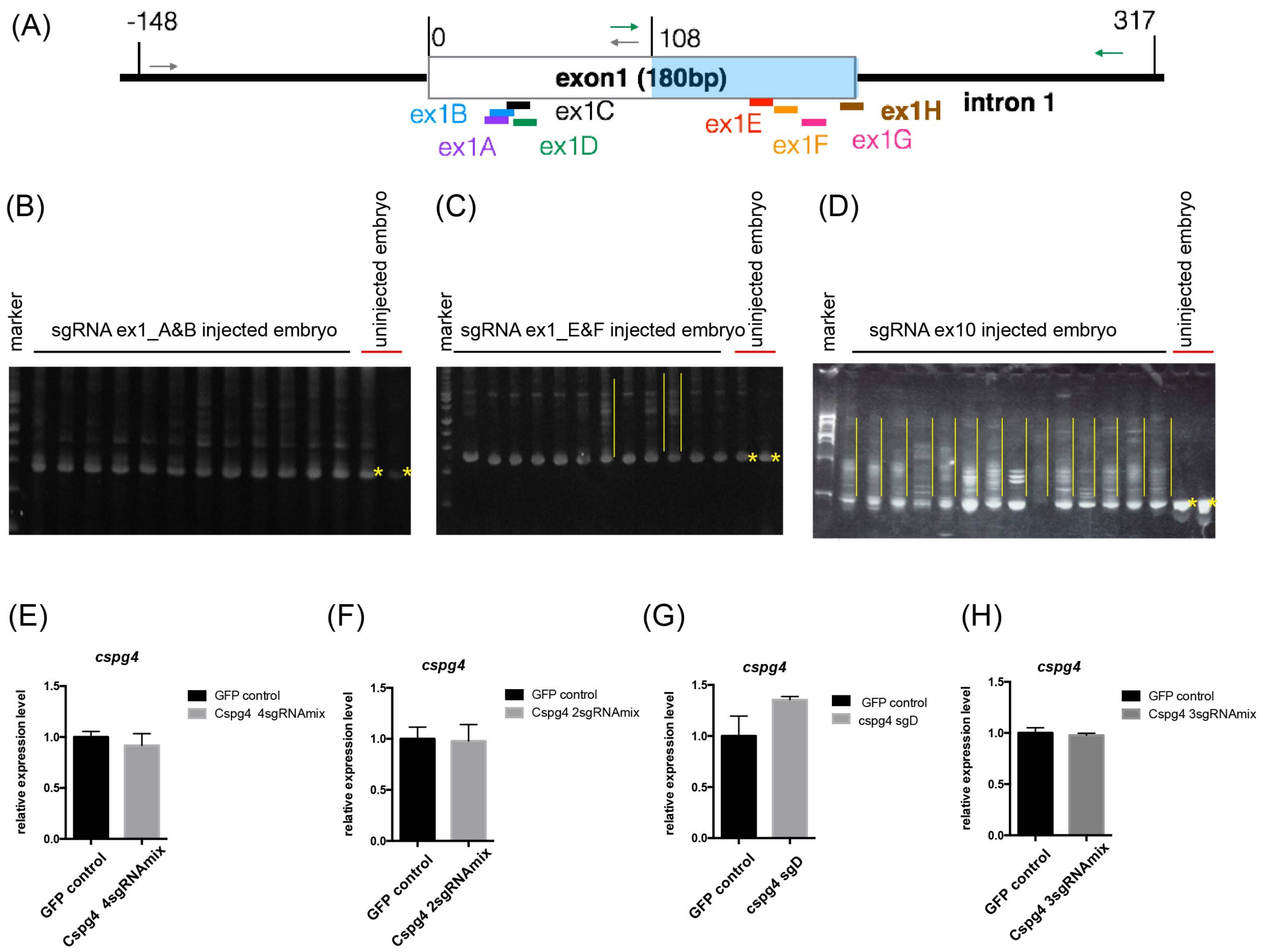

S1_Figure1. The efficiency of sgRNAs targeting the first exon is lower than the sgRNA targeting the tenth exon. (A) A scheme demonstrates the sgRNA target sites in the first exon. Blue box indicates the coding sequence. (B) The genome insertions and deletions (indels) of embryos injected with the sgRNA *cspg4*_sg_ex1A and *cspg4*_sg_ex1B were detected by HMA profile. (C) Indels of embryos injected with the sgRNA *cspg4*_sg_ex1E and *cspg4*_sg_ex1F were detected by HMA profile. (D) Indels of embryos injected with the sgRNA *cspg4*_sg_ex10 were detected by HMA profile. (E) *Cspg4* expression level of embryo injected with dCas9 and four sgRNAs (*cspg4*_sg_ex1A, *cspg4*_sg_ex1B, *cspg4*_sg_ex1E and *cspg4*_sg_ex1F) was detected by quantitative PCR. (F) *Cspg4* expression level of embryo injected with dCas9 and two sgRNAs (*cspg4*_sg_ex1A and *cspg4*_sg_ex1E) was detected by quantitative PCR. (G) *Cspg4* expression level of embryo injected with dCas9 and a sgRNA (*cspg4*_sg_ex1D) was detected by quantitative PCR. (H) *Cspg4* expression level of embryo injected with dCas9 and three sgRNAs (*cspg4*_sg_ex1C, *cspg4*_sg_ex1G, and *cspg4*_sg_ex1H) was detected by quantitative PCR. Embryos injected with sgRNA targeting GFP sequence were used as control group. Gray arrows in panel (A) indicate the primer pair used in the HMA of panel (B). Green arrows in panel (A) indicate the primer pair used in the HMA of panel (C). Yellow asterisks (*) indicate the expected homoduplex bands. The multiple heteroduplex bands labeled by yellow line demonstrated the indels of *cspg4* sgRNAs injected embryos. The significant difference in panel (E, F, G and H) was tested by Student's *t*-test.
