# Supplementary material for: Chondroitin sulfate proteoglycan 4 regulates zebrafish body axis organization via Wnt/planar cell polarity pathway
Source: PLoS One. 2020 Apr 2;15(4):e0230943. doi: 10.1371/journal.pone.0230943 (PMC7117731; doi:10.1371/journal.pone.0230943)
Supplement: S1 Fig — Cspg4em1twu0405 had 275 bp insertion and the insertion sequences including 6 base pairs repeats sequence of the 6586th to 6591th bp of wild-type cspg4 cDNA sequence (labeled by yellow box) and 159 bp repeat sequence from its 6116th bp to the 6274th bp of cspg4 cDNA sequence (labeled by green box) and 110 bp insertion sequence identical to the 6476th bp to the 6585th bp of cspg4 cDNA. gRNA target sequence was indicated by lower case. (PDF) [file pone.0230943.s002.pdf]

```
ENSDART00000112782  6091 TCACACAGAAGCAAATCAAGCAGGGAAGAGTAAGTGTCCGATTTGTGAAGTCCACCTCCCCAAGAGACAG 6160
cspg4em1twu0405     6091 TCACACAGAAGCAAATCAAGCAGGGAAGAGTAAGTGTCCGATTTGTGAAGTCCACCTCCCCAAGAGACAG 6160

ENSDART00000112782  6161 CGTGGCCTTTGTTGCTCGAAGCAGGGCAGCGAATGTATCCTCAGTCCTCAACATTACTGTGAAACCACTG 6230
cspg4em1twu0405     6161 CGTGGCCTTTGTTGCTCGAAGCAGGGCAGCGAATGTATCCTCAGTCCTCAACATTACTGTGAAACCACTG 6230

ENSDART00000112782  6231 GCAAAGGTGGCACAAAATCCCCTGCTACCCAGAGGTGCTACTGTGCTTGTAGACAGAAAGTTGTTGGACG 6300
cspg4em1twu0405     6231 GCAAAGGTGGCACAAAATCCCCTGCTACCCAGAGGTGCTACTGTGCTTGTAGACAGAAAGTTGTTGGACG 6300

ENSDART00000112782  6301 CTACTCCTCTGGCCAACAAAACCAGAACATCTCCAACCTTCAGCATGATTCAACAACCTCAAGGAGCTCG 6370
cspg4em1twu0405     6301 CTACTCCTCTGGCCAACAAAACCAGAACATCTCCAACCTTCAGCATGATTCAACAACCTCAAGGAGCTCG 6370

ENSDART00000112782  6371 GTTTGTCAAAAGGGGTGGGCTCGATGACGGACAACCAGTGAGCTCTTTCACTCAGCAGGATCTGGATGAA 6440
cspg4em1twu0405     6371 GTTTGTCAAAAGGGGTGGGCTCGATGACGGACAACCAGTGAGCTCTTTCACTCAGCAGGATCTGGATGAA 6440

ENSDART00000112782  6441 GGCCGGGTAGCTTTggagatcttaaacacaaccgggGCCAAAACGGTGGAGGTCAAAATCAAGATGAAG 6510
cspg4em1twu0405     6441 GGCCGGGTAGCTTTggagatcttaaacacaaccgggGCCAAAACGGTGGAGGTCAAAATCAAGATGAAG 6510

ENSDART00000112782  6511 CACGGTTTCTCCTAAAGGCGCATGGCGTACCTCCTGCAGAATGTGTCTTGCCCTTCCACGTGGTTCCTTA 6580
cspg4em1twu0405     6511 CACGGTTTCTCCTAAAGGCGCATGGCGTACCTCCTGCAGAATGTGTCTTGCCCTTCCACGTGGTTCCTTA 6580

ENSDART00000112782  6581 TGATC----------------------------------------------------------------- 6585
cspg4em1twu0405     6581 TGATCCTTCCAAAGAGTAAGTGTCCGATTTGTGAAGTCCACCTCCCCAAGAGACAGCGTGGCCTTTGTTG 6650

ENSDART00000112782  6586 ---------------------------------------------------------------------- 6586
cspg4em1twu0405     6651 CTCGAAGCAGGGCAGCGAATGTATCCTCAGTCCTCAACATTACTGTGAAACCACTGGCAAAGGTGGCACA 6720

ENSDART00000112782  6586 ---------------------------------------------------------------------- 6586
cspg4em1twu0405     6721 AAATCCCCTGCTACCCAGAGGTGCTACTGTGgGCCAAAACGGTGGAGGTCAAAATCAAGATGAAGCACGG 6790

ENSDART00000112782  6586 ---------------------------------------------------------------------- 6586
cspg4em1twu0405     6791 TTTCTCCTAAAGGCGCATGGCGTACCTCCTGCAGAATGTGTCTTGCCCTTCCACGTGGTTCCTTATGATC 6860

ENSDART00000112782  6586 CTTCCAAAGTTTATGGAGCCACATTGCTCAAGGTGCCACCGGTGTCGGTTTCTGACAGCAATGAAGCAGG 6655
cspg4em1twu0405     6861 CTTCCAAAGTTTATGGAGCCACATTGCTCAAGGTGCCACCGGTGTCGGTTTCTGACAGCAATGAAGCAGG 6930
```

S2_Fig1. The DNA sequence of transmembrane domain mutant line *cspg4^{em1twu0405}* was aligned to the wild-type *cspg4* cDNA sequence (ENSDART00000112782). *cspg4^{em1twu0405}* had 275 bp insertion and the insertion sequences including 6 base pairs repeats sequence of the 6586th to 6590th bp of wild-type *cspg4* cDNA sequence (labeled by yellow box) and 159 bp repeat sequence from its 6116th bp to the 6274th bp of *cspg4* cDNA sequence (labeled by green box) and 110 bp insertion sequence identical to the 6476th bp to the 6585th bp of *cspg4* cDNA (labeled by blue box). gRNA target sequence was indicated by lower case.
