# Supplementary material for: Chondroitin sulfate proteoglycan 4 regulates zebrafish body axis organization via Wnt/planar cell polarity pathway
Source: PLoS One. 2020 Apr 2;15(4):e0230943. doi: 10.1371/journal.pone.0230943 (PMC7117731; doi:10.1371/journal.pone.0230943)
Supplement: S2 Fig — The body length at 1 dpf (A) and the angle between anterior end and tailbud at tailed stage. (B) were not rescued by co-injecting pdgfaa mRNA. (PDF) [file pone.0230943.s003.pdf]

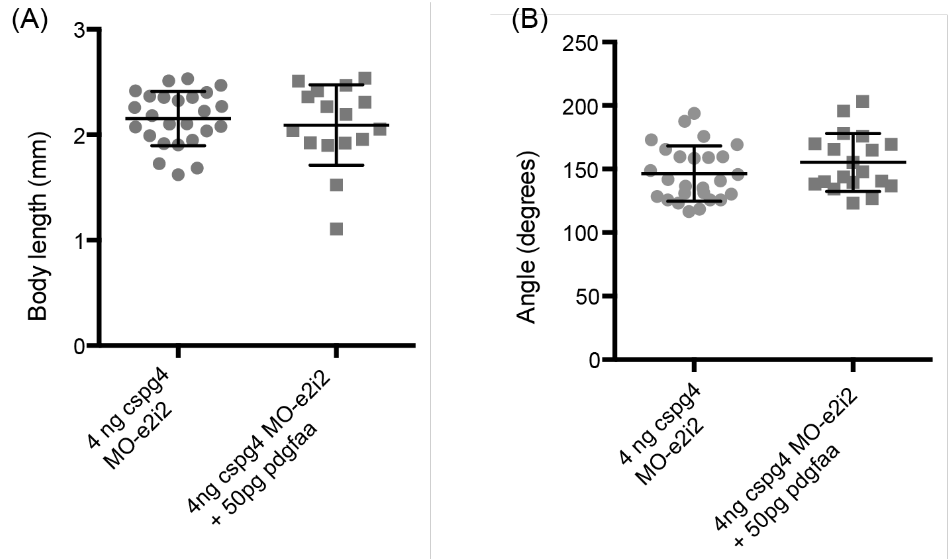

S2_Fig2. The phenotypes of *cspg4* morphants could not be rescued by co-injecting *pdgfaa* mRNA. The body length at 1 dpf (A) and the angle between anterior end and tailbud at tailed stage(B) were not rescued by co-injecting *pdgfaa* mRNA.
