# Supplementary figures and images for: Chondroitin sulfate proteoglycan 4 regulates zebrafish body axis organization via Wnt/planar cell polarity pathway
Source: PLoS One. 2020 Apr 2;15(4):e0230943. doi: 10.1371/journal.pone.0230943 (PMC7117731; doi:10.1371/journal.pone.0230943)

Fig2A

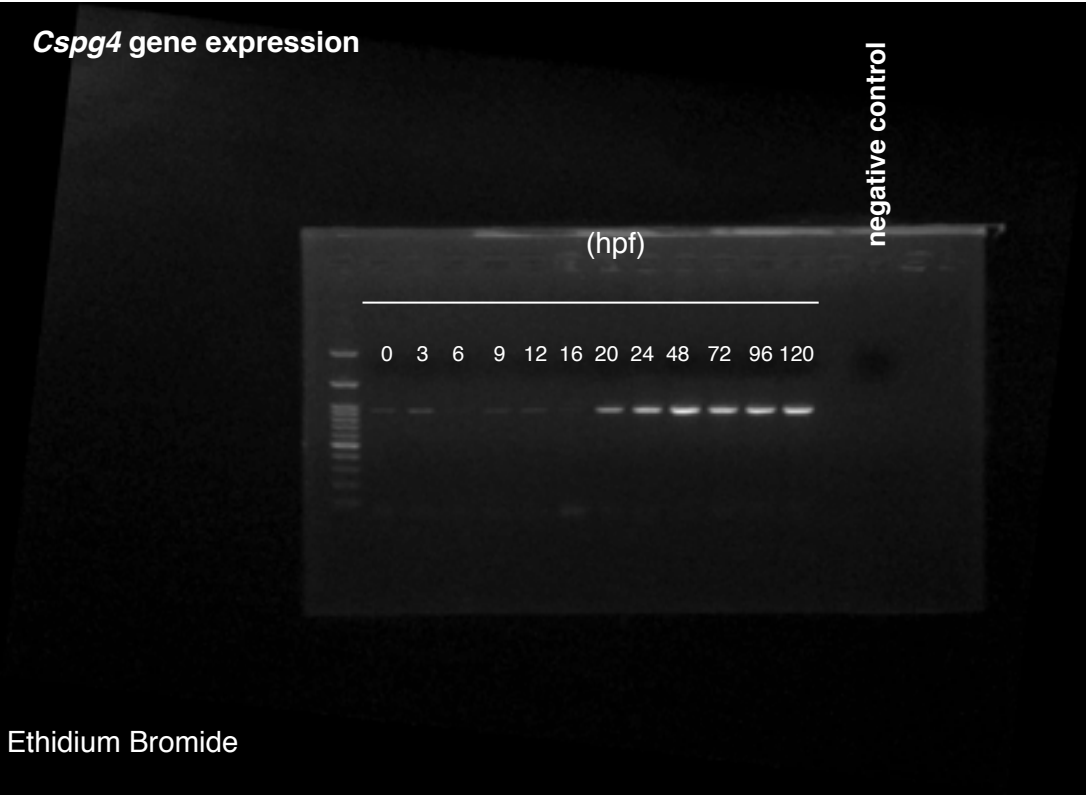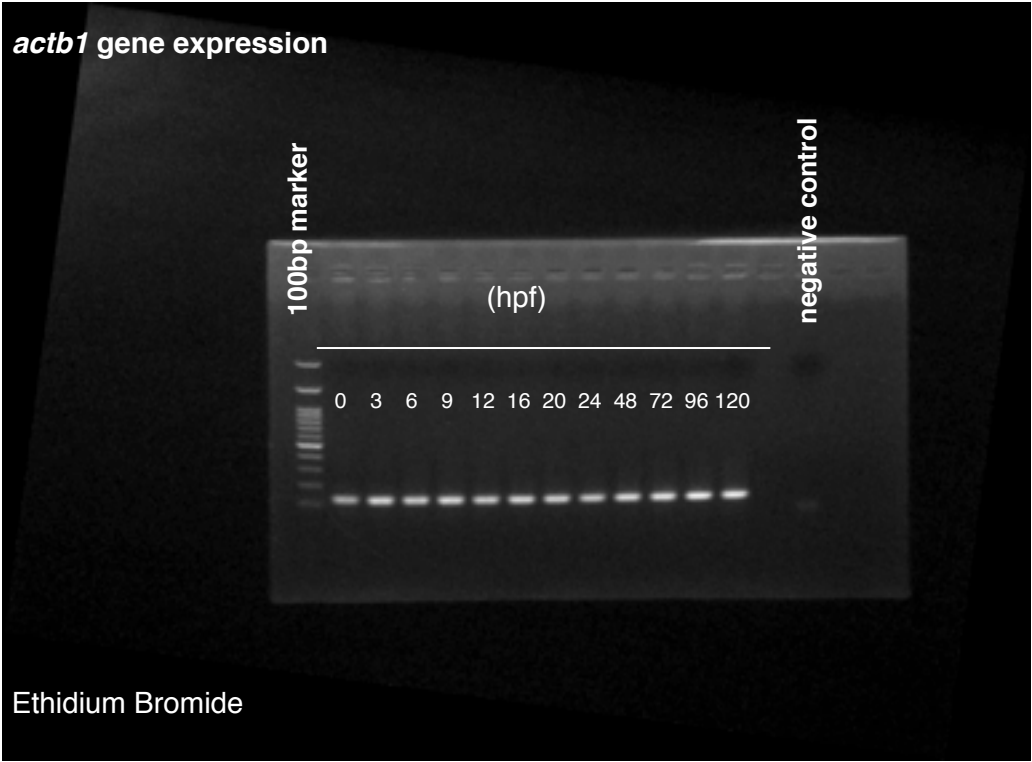

Fig3A

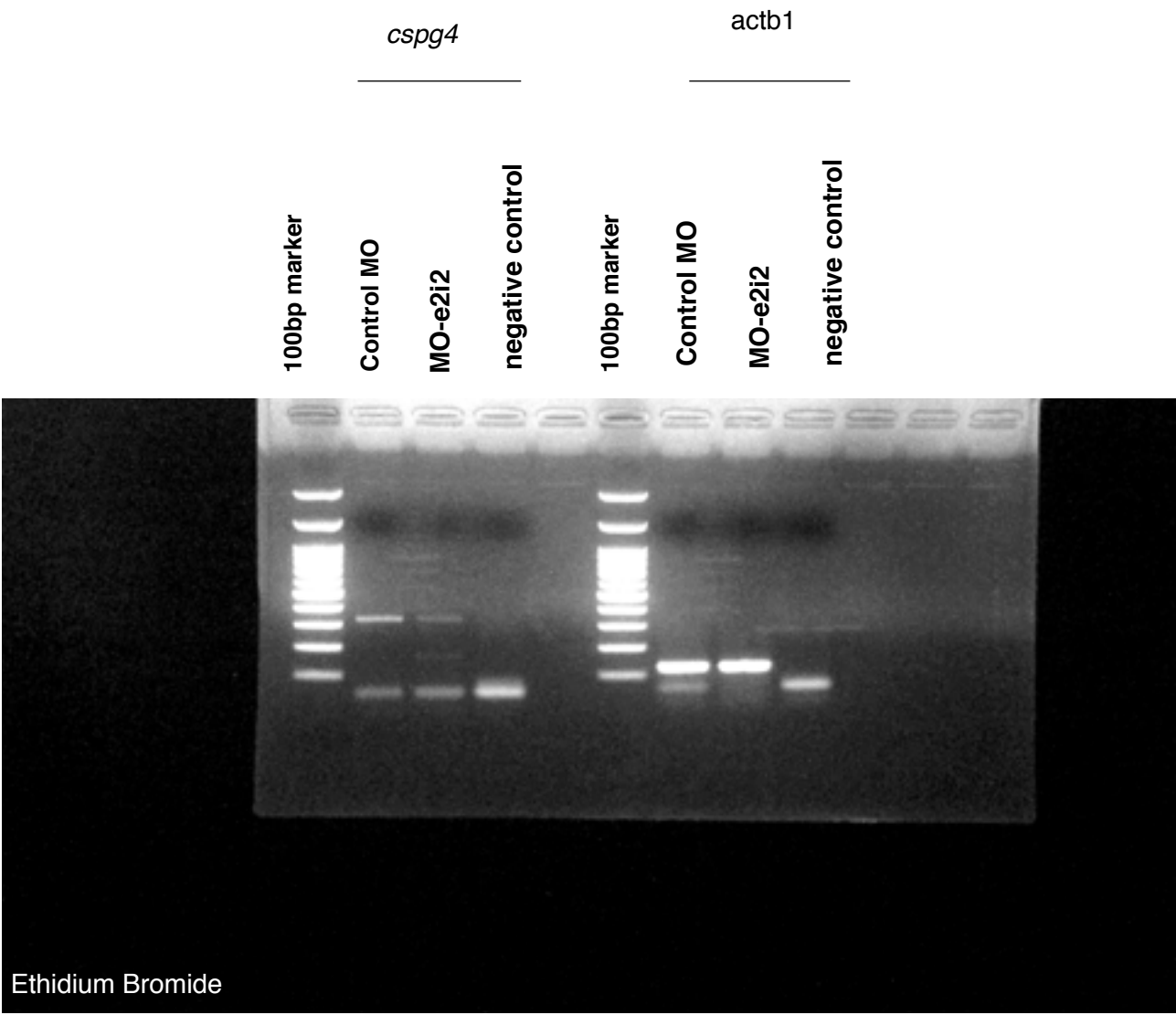

Fig6B

IB:Flag

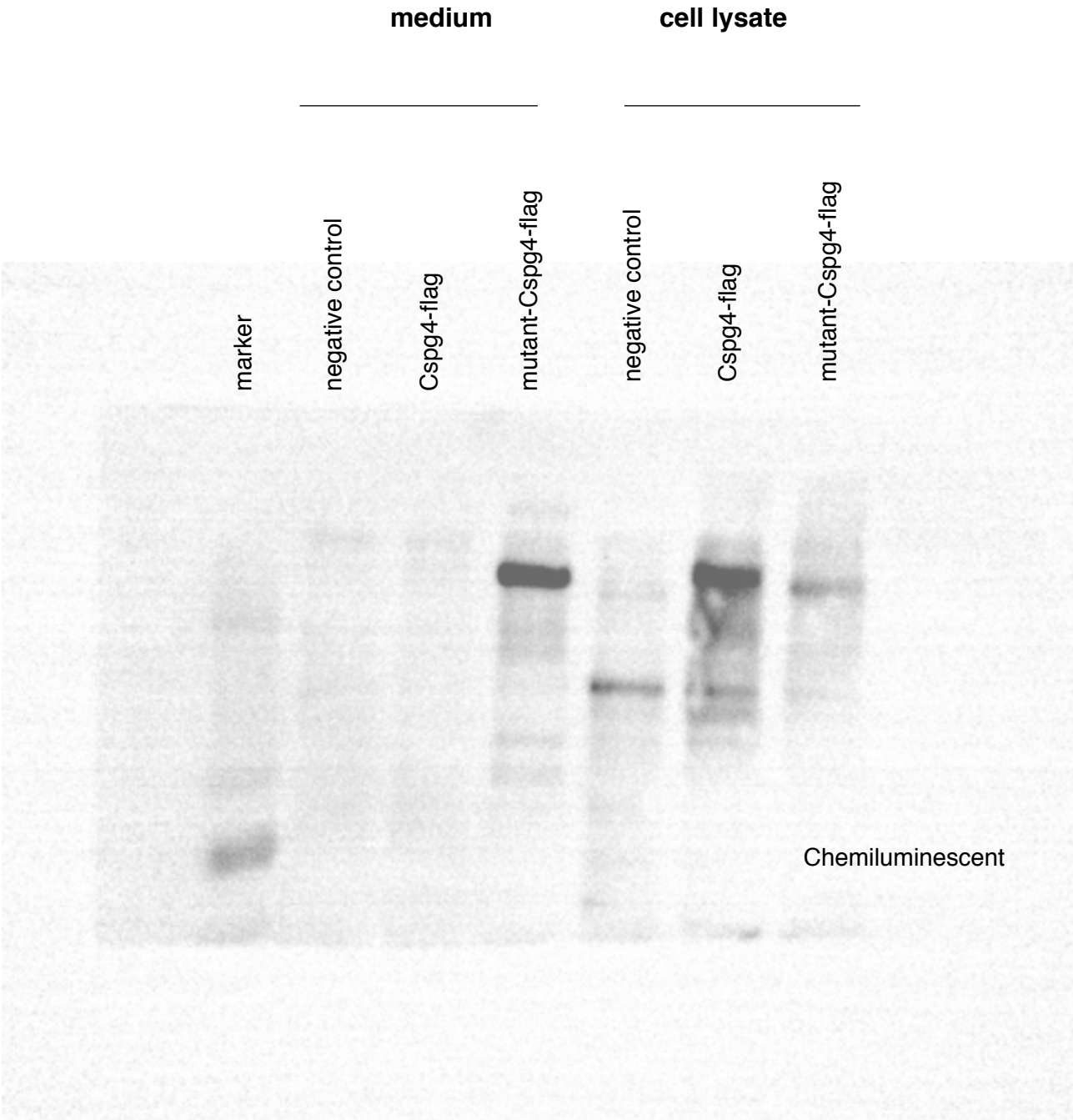

Flg7A  
IB: Flag

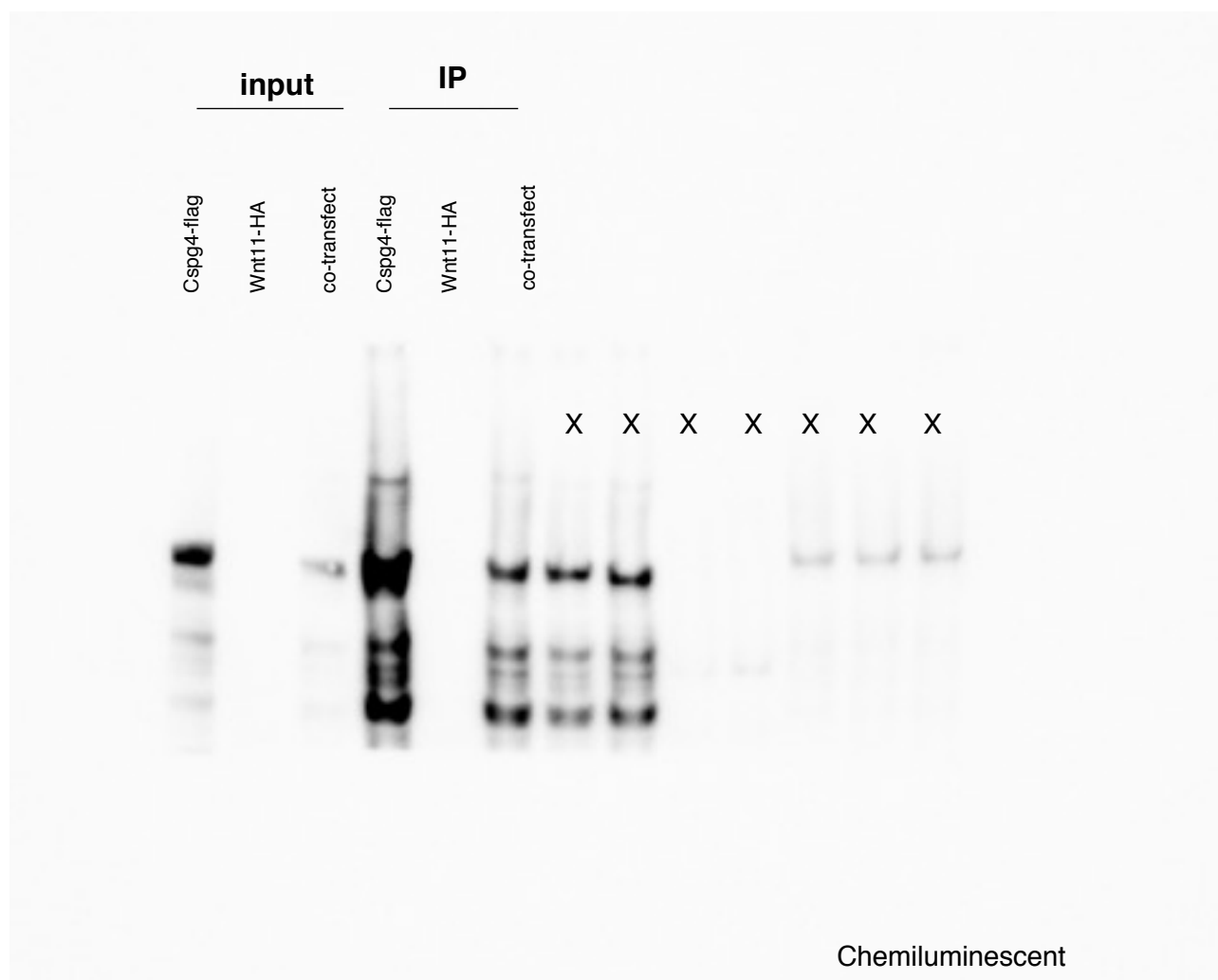

x: lanes not included in the final figure

Fig. 7B  
IB:HA

input

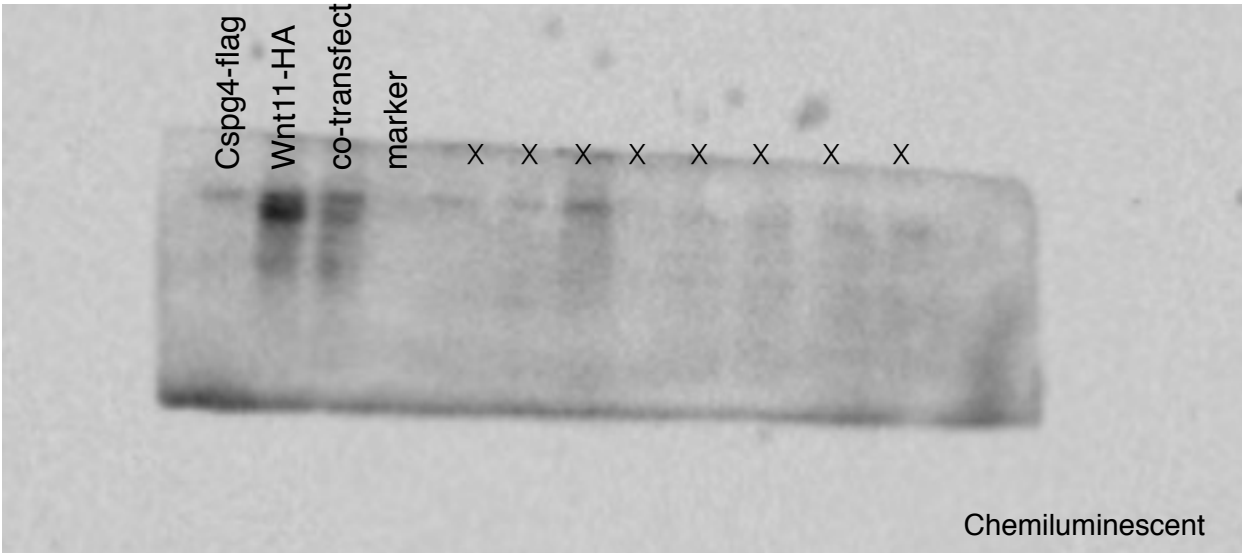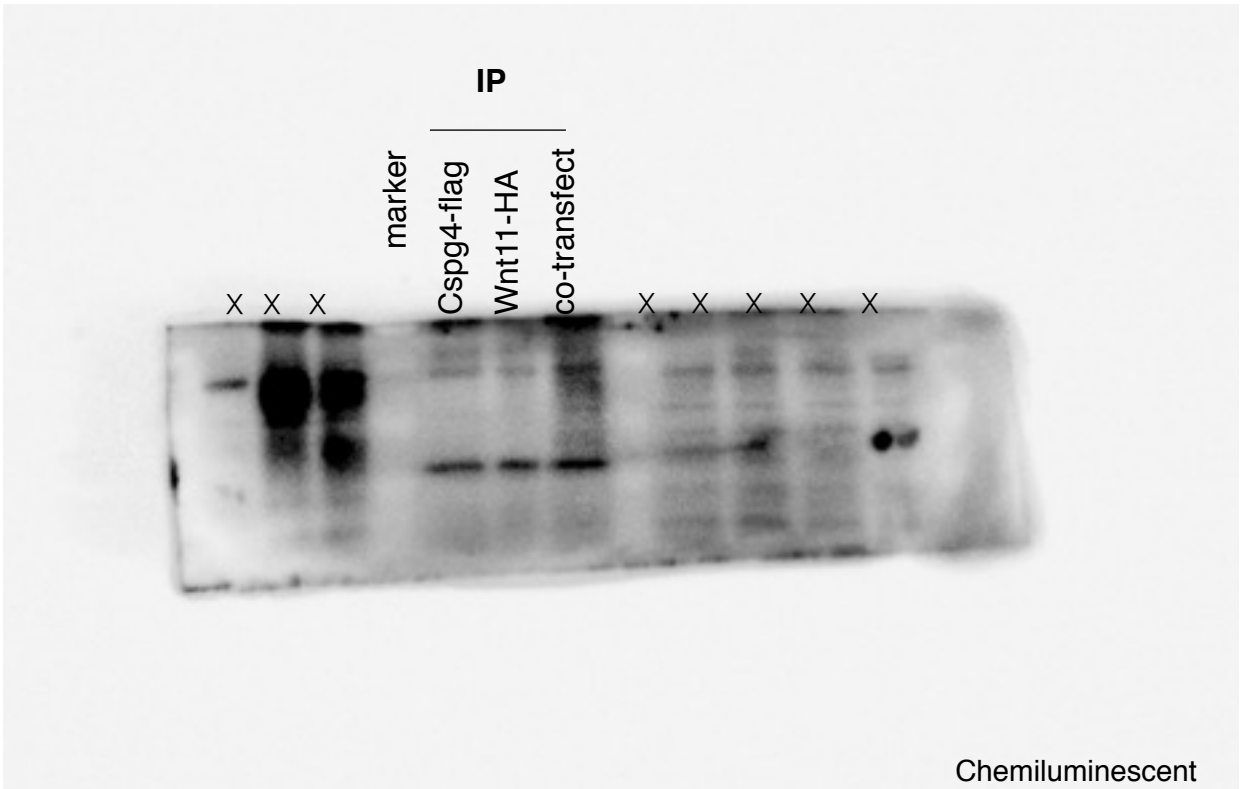

Supplement: S1 Raw Images — (PDF) [file pone.0230943.s004.pdf]
